# Supplementary material for: Realization of fermionic Laughlin state on a quantum processor
Source: Nat Commun. 2026 Jun 8;17:4919. doi: 10.1038/s41467-026-72769-y (PMC13247042; doi:10.1038/s41467-026-72769-y)
Supplement: Supplementary file 1 — Supplementary Information [file 41467_2026_72769_MOESM1_ESM.pdf]

# Supplementary Information: Realization of fermionic Laughlin state on a quantum processor

Lingnan Shen,[1] Mao Lin,[2] Cedric Yen-Yu Lin,[2] Di Xiao*,[3,1,4] and Ting Cao†[3]
[1] *Department of Physics, University of Washington, Seattle, WA, USA*
[2] *Amazon Braket, Seattle, WA, USA*
[3] *Department of Material Science and Engineering, University of Washington, Seattle, WA, USA*
[4] *Pacific Northwest National Laboratory, Richland, WA, USA*
(Dated: April 17, 2026)

* Correspondence to: dixiao@uw.edu
† Correspondence to: tingcao@uw.edu

**CONTENTS**

Supplementary Note 1 – Hamiltonian under the Tao-Thouless limit S1
A. Krylov subspace formation S2
B. Entanglement entropy S2

Supplementary Note 2 – Additional results of Hamiltonian variational ansatz optimization S2
A. Optimizing Hamiltonian variational ansatz correspond to $H_{\mathrm{TT}}$ S2
B. Optimizing $|\psi(\{\beta_j\})\rangle_{\mathrm{eff}}$ at $N_e = 8$ S3
C. Optimization result for ansatz with $V_{40}$ term S3

Supplementary Note 3 – Detailed resource required by Hamiltonian variational ansatz circuit S3
A. Example Hamiltonian variational ansatz circuit for $N_e = 6$ S3
B. Circuit gate count and depth scaling S3

Supplementary Note 4 – Measurement of topological entanglement entropy S4
A. Extracting topological entanglement entropy $\gamma_{\mathrm{topo}}$ through geometric deformation S5
B. Randomized measurement protocol for second-order Rényi entropy S5

Supplementary Note 5 – Quantum hardware data S6
A. IonQ Aria-1 data S6
B. IonQ Aria-1 debiasing data S6
C. Symmetry-verification post-selection S7
D. IonQ Forte-1 extended data of second Rényi entropy measurement S8

References S9

## Supplementary Note 1 – Hamiltonian under the Tao-Thouless limit

The system Hamiltonian is given by

$$H = \sum_j \sum_{k>m} V_{km} c^\dagger_{j+m} c^\dagger_{j+k} c_{j+k+m} c_j, \tag{1}$$

with the Haldane-Trugman-Kivelson psuedopotential matrix element

$$V_{km} = \frac{16\pi^2}{L_y}(k^2 - m^2) e^{-\frac{2\pi^2(k^2+m^2)}{L_y^2}}. \tag{2}$$

In this formulation, the scattering terms characterized by nonzero values of $m$ ($m \neq 0$) become exponentially suppressed compared to the dominant electrostatic interaction terms ($V_{k0}$) in the Tao-Thouless limit $L_y \to 0$. Thus the system becomes a charge-density-wave (CDW) state $|\Psi_{\mathrm{CDW}}\rangle = |100100100...\rangle$.

The lowest order approximation beyond the Tao-Thouless (TT) limit is made by truncating long-range interactions up to $(k+m) \leq 3$ terms, yielding the TT limit Hamiltonian

$$H_{\mathrm{TT}} = \sum_j [V_{10}\hat{n}_j\hat{n}_{j+1} + V_{20}\hat{n}_j\hat{n}_{j+2} + V_{30}\hat{n}_j\hat{n}_{j+3} + (V_{21}c^\dagger_{j+1}c^\dagger_{j+2}c_{j+3}c_j + \mathrm{H.c.})], \tag{3}$$

which includes only the lowest-order scattering term $V_{21}c^\dagger_{j+1}c^\dagger_{j+2}c_{j+3}c_j$.

### A. Krylov subspace formation

The fidelity decay at isotropic limit for $H_{\mathrm{TT}}$ with $(k+m \leq 3)$ originates from Hilbert space fragmentation, where the system is confined to a Krylov subspace $\mathcal{K}$ defined as

$$\mathcal{K} \equiv \mathrm{Span}\{|\Psi_0\rangle, H_{\mathrm{TT}}|\Psi_0\rangle, H^2_{\mathrm{TT}}|\Psi_0\rangle, ...\}, \tag{4}$$

the subspace connected to the root charge-density-wave state $|\Psi_0\rangle = |\Psi_{\mathrm{CDW}}\rangle = |100100100...\rangle$ by the action of $H_{\mathrm{TT}}$. This Krylov subspace $\mathcal{K}$ is significantly *smaller* than the full Hilbert space of Laughlin state

$$\mathcal{H}_{\mathrm{TT}} = \mathcal{K} \subset \mathcal{H}_{\mathrm{Laughlin}},\ \dim(\mathcal{K}) \ll \dim(\mathcal{H}_{\mathrm{Laughlin}}). \tag{5}$$

Thus, $H_{\mathrm{TT}}$ remains valid only near the TT limit and fails to capture the relevant correlations in the full Laughlin state and its underlying topology. Including higher-order scatterings like $V_{31}$ in $H_{\mathrm{eff}}$ breaks the constraint of $\mathcal{K}$, connecting the entire Hilbert space with $|\Psi_0\rangle$ and ensuring the expressiveness of the Hamiltonian variational ansatz.

### B. Entanglement entropy

We further analyze the validity of different truncation range by comparing the Rényi entanglement entropy $S_A$ of their ground states. The second-order Rényi entanglement entropy is defined as

$$S^{(2)}_A = -\ln \mathrm{Tr}\rho^2_A, \tag{6}$$

where $\rho_A = \mathrm{Tr}_B(|\Psi\rangle\langle\Psi|)$ is the reduced density matrix for subsystem A by tracing over the degrees of freedom of subsystem B. In the following calculation, we choose $A$ to be the left half orbitals of the cylinder.

For a system in $d$ dimensions with a finite correlation length $l$, the entanglement entropy satisfies the area law

$$S_A \simeq \alpha L^{d-1}, \tag{7}$$

where $L$ is the length of the boundary between the two blocks. For the two-dimensional Laughlin state, we expect $S_A$ to scale linearly with the cylinder's circumference $L_y$.

As shown in Supplementary Figure 1, in the TT limit ($L_y \to 0$), the system ground state approaches the charge-density-wave state $|\Psi_{\mathrm{CDW}}\rangle = |100100100...\rangle$ which is a product state, leading to $S_A \to 0$. In the region $L_y \lesssim 7$, the entanglement entropy of the ground state for both interaction truncation $(k+m \leq 3, 4)$ align closely. However, beyond $L_y > 7$, the entropy of the ground state of $H_{\mathrm{TT}}$ quickly saturates, deviating from the expected area-law scaling of a genuine fractional quantum Hall liquid. This indicates that $H_{\mathrm{TT}}$ fails to capture relevant entanglement in the full Laughlin state and its underlying topology. In contrast, extending the truncation range to $(k+m \leq 4)$ recovers the expected area law behavior for an incompressible topological quantum liquid.

## Supplementary Note 2 – Additional results of Hamiltonian variational ansatz optimization

### A. Optimizing Hamiltonian variational ansatz correspond to $H_{\mathrm{TT}}$

In addition, we optimized the Hamiltonian variational ansatz correspond to the Tao-Thouless limit Hamiltonian $H_{\mathrm{TT}}$ in Supplementary Equation (3) for system size $N_e = 6$

$$|\psi(\{\beta_j\})\rangle_{\mathrm{TT}} = \hat{U}_{20}\hat{U}_{10}\hat{U}_{30}\hat{U}_{21}|\Psi_{\mathrm{CDW}}\rangle. \tag{8}$$

The highest fidelity achieved was $\mathcal{F} = 0.79$, significantly lower than the $\mathcal{F} = 0.93$ obtained for $|\psi(\beta_j)\rangle_{\mathrm{eff}}$ in the main text.

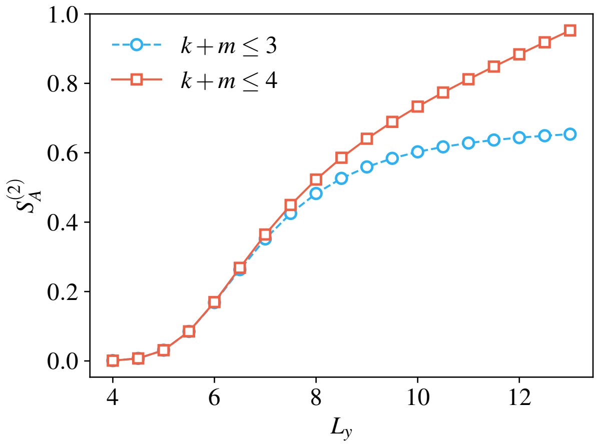


Supplementary Figure 1. Second Rényi entanglement entropy $S_A^{(2)}$ of the ground state of the effective Hamiltonian for various truncation ranges of interactions ($k+m \leq 3,4$) on a finite cylinder geometry, as a function of cylinder circumference $L_y$. The system size is $N_e = 8$, and the bipartition is chosen to divide the system into two equal halves, each containing 11 orbitals.

### B. Optimizing $|\psi(\{\beta_j\})\rangle_{\mathbf{eff}}$ at $N_e = 8$

We also optimized the Hamiltonian variational ansatz $|\psi(\beta_j)\rangle_{\text{eff}}$, as defined in the main text, for larger system size $N_e = 8$. The highest fidelity achieved through direct optimization was $\mathcal{F} = 0.88$. Remarkably, this optimized fidelity closely matches the fidelity obtained by extrapolating parameters previously optimized at $N_e = 6$ to larger system size $N_e = 8$.

### C. Optimization result for ansatz with $V_{40}$ term

We included an additional term $\hat{U}_{40}$ and performed optimization at system size $N_e = 6$. Specifically, we considered the variational ansatz:

$$|\psi(\{\beta_j\})\rangle_{\text{TT}} = \hat{U}_{40}\hat{U}_{20}\hat{U}_{10}\hat{U}_{30}\hat{U}_{21}\,|\Psi_{\text{CDW}}\rangle\,. \tag{9}$$

Upon optimization, the maximum fidelity achieved was $\mathcal{F} = 0.929$, only marginally improved compared to the ansatz without the $\hat{U}_{40}$ term ($\mathcal{F} = 0.927$). Thus the $H_{\text{eff}}$ defined in main text represent the minimum effective Hamiltonian for constructing the HVA for quantum simulation of $\nu = 1/3$ Laughlin state.

## Supplementary Note 3 – Detailed resource required by Hamiltonian variational ansatz circuit

### A. Example Hamiltonian variational ansatz circuit for $N_e = 6$

We present an example quantum circuit for a 16-qubit system, corresponding to $N_e = 6$, which was executed on IonQ's Aria-1 trapped-ion quantum processor.

### B. Circuit gate count and depth scaling

Here, we analyze how the circuit two-qubit (CNOT) gate count and depth scale with both the interaction truncation of the Hamiltonian and the system size. The Hamiltonian variational ansatz constructed with respect to Eq. 1 is

$$|\psi\rangle = \prod_{k>m} \hat{U}_{km}|\Psi_{\text{CDW}}\rangle, \quad \hat{U}_{km} = \prod_{j} \exp\left[-i\beta_{km}(c^\dagger_{j+m}c^\dagger_{j+k}c_{j+k+m}c_j + \text{H.c.})\right], \tag{10}$$

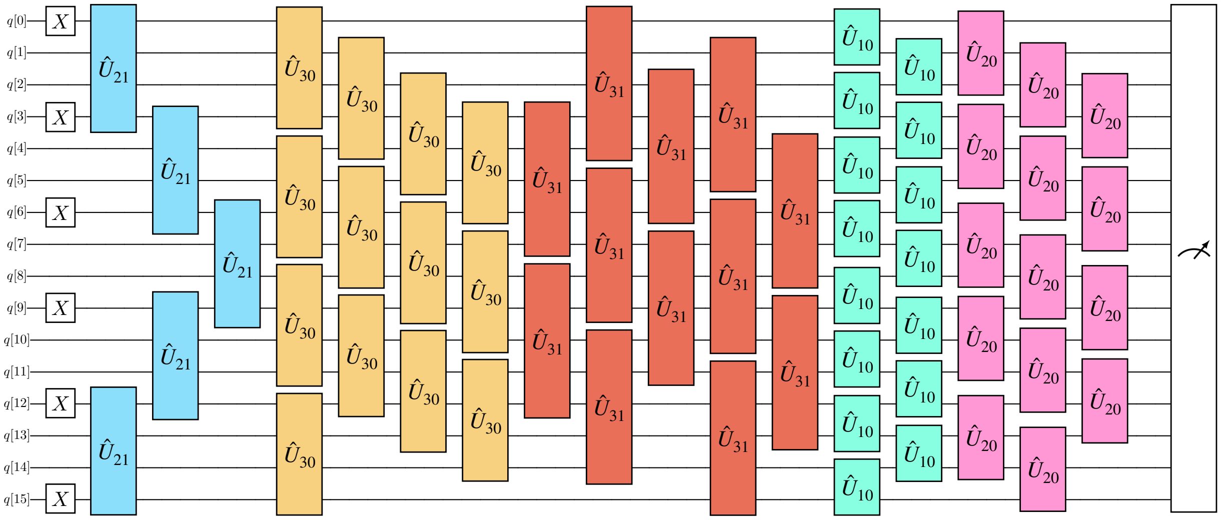


Supplementary Figure 2. Example circuit for the 16-qubit HVA corresponding to the $\nu = 1/3$ Laughlin state for system size $N_e = 6$.

where the interaction truncation range is defined by $k + m \leq$ cutoff. The CNOT gate count (Supplementary Table 1) grows roughly linearly with system size and exponentially with the cutoff while the CNOT depth (Supplementary Table 2) scales approximately exponentially with the truncation cutoff and only weakly with system size. We show in Supplementary Table 3 the quantum resources needed for a single repetition of the HVA we constructed in main text.

| Cutoff | $N_e = 6$ | $N_e = 8$ | $N_e = 10$ | $N_e = 12$ |
|---|---|---|---|---|
| 3 | 305 | 443 | 581 | 719 |
| 4 | 533 | 785 | 1037 | 1289 |
| 5 | 1156 | 1752 | 2335 | 2929 |
| 6 | 1722 | 2660 | 3585 | 4516 |
| 7 | 2775 | 4411 | 6035 | 7675 |
| 8 | 3714 | 6048 | 8375 | 10713 |
| 9 | 5053 | 8537 | 11999 | 15482 |
| 10 | 6081 | 10593 | 15076 | 19591 |

Supplementary Table 1. CNOT gate count with respect to interaction range cutoff and system size $N_e$.

| Cutoff | $N_e = 6$ | $N_e = 8$ | $N_e = 10$ | $N_e = 12$ |
|---|---|---|---|---|
| 3 | 86 | 86 | 86 | 86 |
| 4 | 177 | 179 | 181 | 181 |
| 5 | 507 | 515 | 515 | 520 |
| 6 | 892 | 906 | 906 | 912 |
| 7 | 1805 | 1820 | 1821 | 1832 |
| 8 | 2722 | 2848 | 2851 | 2860 |
| 9 | 4031 | 4722 | 4722 | 4729 |
| 10 | 5039 | 6564 | 6560 | 6576 |

Supplementary Table 2. CNOT depth with respect to interaction range cutoff and system size $N_e$.

## Supplementary Note 4 – Measurement of topological entanglement entropy

| $N_e$ | Qubits | CNOT gates |
|---|---|---|
| 6 | 16 | 369 |
| 8 | 22 | 543 |
| 10 | 28 | 711 |
| 12 | 34 | 883 |

Supplementary Table 3. Number of qubits and CNOT gates for a single repetition of our HVA $|\psi(\{\beta_j\})\rangle_{\text{eff}}$ for various system size $N_e$. The number scales approximately linearly with the system size.

### A. Extracting topological entanglement entropy $\gamma_{\text{topo}}$ through geometric deformation

To isolate the topological contribution to the entanglement, we analyze the scaling behavior of the second-order Rényi entropy $S_A^{(2)}$ of the variationally optimized ground state $|\psi(\{\beta_j\})\rangle_{\text{eff}}$ using the relation

$$S_A^{(2)} \simeq \alpha L_y - \gamma_{\text{topo}}, \tag{11}$$

where $\alpha$ is a non-universal constant and $L_y$ is the circumference of the cylinder. Since the $\nu = 1/3$ Laughlin phase is gapped, its ground state correlations decay exponentially and the entanglement associated with an orbital cut is localized within a finite correlation length around each entanglement boundary. Previous analysis on the cylinder give a bulk correlation length $\zeta_{1/3} \approx 1.38\,\ell_B$ for the Laughlin state [1], which in the Landau-gauge orbital basis corresponds to an orbital correlation length $\xi_{\text{orb}} \equiv \zeta/\Delta x \sim (\zeta/\ell_B)(L_y/2\pi\ell_B)$ (with $\Delta x = 2\pi\ell_B^2/L_y$), i.e. $\xi_{\text{orb}} \leq 3$ orbitals over our experimental near-isotropic regime of $L_y$. Accordingly, we choose the subsystem $A$ (containing $N_A = 6$ orbitals) around the bulk region of the cylinder, see Supplementary Note 5 Sec. D for details, that is larger than $\xi_{\text{orb}}$ while keeping the randomized-measurement sampling cost manageable (as the number of measurements required to estimate purities grows exponentially with subsystem size [2]). This introduces two disjoint entanglement boundaries between subsystem $A$ and the environment $B$. Consequently, the topological correction is doubled, leading to a theoretical prediction of: $-\gamma_{\text{topo}} = -2\ln\sqrt{3} \approx 1.10$.

To benchmark our variational ansatz, we optimized $|\psi(\{\beta_j\})\rangle_{\text{eff}}$ for $N_e = 6$ electrons on a classical simulator across a range of cylinder circumferences $L_y \in [5.5, 10.0]$. We computed $S_A^{(2)}$ for each optimized state and performed a linear fit to the area law scaling form. The extracted intercept from the classical simulation is $-\gamma_{\text{vqe}} = -1.09$ by bootstrap refitting over three system partitions' entropy (see Supplementary Note 5 for detail), which stands in excellent agreement with the theoretical prediction, validating the capability of the ansatz to capture the essential topological correlations of the Laughlin phase.

To verify that the entanglement entropy obtained from $|\psi(\{\beta_j\})\rangle_{\text{eff}}$ arises from intrinsic topological order rather than simple density modulation, we variationally optimized a minimal excitation-preserving ansatz constrained to match only the local density $\langle n_j\rangle$ profile as obtained from $|\psi(\{\beta_j\})\rangle_{\text{eff}}$ at each $L_y$. Despite achieving a density profile of $\max_j \Delta\langle n_j\rangle < 0.05$ from $|\psi(\{\beta_j\})\rangle_{\text{eff}}$, the minimal excitation-preserving ansatz exhibits a significantly smaller $S_A^{(2)}$.

### B. Randomized measurement protocol for second-order Rényi entropy

To experimentally access the topological entanglement entropy, we measured the second-order Rényi entropy $S_A^{(2)} = -\ln \text{Tr}(\rho_A^2)$ of the same six-qubit subsystem $A$. We employed the randomized measurement protocol [2], which estimates the purity $X_A = \text{Tr}(\rho_A^2)$ from the statistical correlations of bitstring outcomes in randomized local bases, avoiding the exponential overhead of full quantum state tomography.

For each cylinder circumference $L_y$ we prepared the optimized HVA and applied an ensemble of $N_U = 200$ independent random unitaries. Each unitary $U^{(r)} = \bigotimes_{i=1}^{N} U_i^{(r)}$ consisted of local single-qubit gates drawn from the Circular Unitary Ensemble. Following the application of $U^{(r)}$, the system was measured in the computational basis, yielding a set of $N_M = 300$ bitstrings per unitary. For a subsystem $A$ of size $N_A$, the estimated purity $X_A^{(r)}$ by a random unitary $U^{(r)}$ is given by:

$$X_A^{(r)} = 2^{N_A} \sum_{s_A, s'_A} (-2)^{-D(s_A, s'_A)} P(s_A) P(s'_A), \tag{12}$$

where $s_A$ and $s'_A$ denote bitstring outcomes restricted to the subsystem $A$, $D(s_A, s'_A)$ is the Hamming distance between them and $P(s_A)$ denotes the probability of observing $s_A$. In practice, $P(s_A)^2$ is a biased estimator for $\mathbb{E}(P(s_A))^2$ due

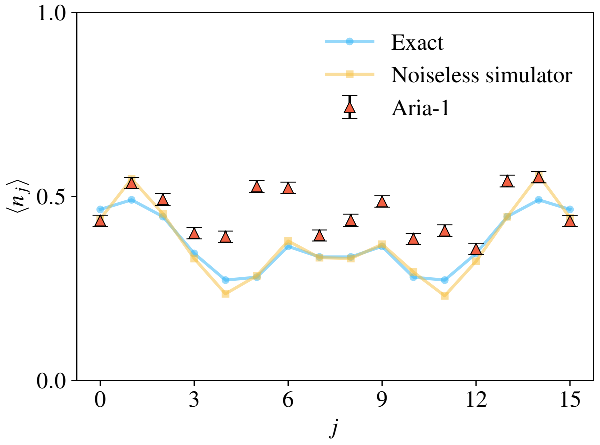


Supplementary Figure 3. Local density $n_j$ at site $j$ for system size $N_e = 6$. Experimental results obtained without error-mitigation effort are compared with noiseless simulation of the optimized HVA (orange square) and exact values from ED (blue circle). Error bars indicate 68% confidence intervals obtained by means of percentile bootstrap.

to finite shot noise. Instead, we calculated the purity using an unbiased estimator

$$X_A^{(r)} = 2^{N_A} \left[ \sum_{s_A \neq s'_A} (-2)^{-D(s_A, s'_A)} \frac{N_M}{N_M - 1} P(s_A)P(s'_A) + \sum_{s_A} \frac{P(s_A)(N_M P(s_A) - 1)}{N_M - 1} \right]. \tag{13}$$

The estimated purity is then acquired by averaging over the unitary ensemble

$$\mathrm{Tr}(\rho_A^2) = \frac{1}{N_U} \sum_{r=1}^{N_U} X_A^{(r)}. \tag{14}$$

In this work, we performed non-parametric bootstrap analysis on the ensemble of $N_U$ purity estimates to determine second Rényi entropy.

To mitigate the effects of hardware drift and fluctuations during execution, we implemented an interleaved execution scheme. Rather than collecting all $N_M$ shots for a given unitary at once, we cycled through the full unitary ensemble in small batches to accumulate the total measurement shots, improving effective stability without changing $N_U$ and $N_M$.

## Supplementary Note 5 – Quantum hardware data

### A. IonQ Aria-1 data

We sampled 1000 shots on IonQ's Aria-1 quantum computer. Experimental results without using any error-mitigation technique are shown for local density $n_j$ (Supplementary Figure 3) and site-averaged correlation $C(d)$ (Supplementary Figure 4).

### B. IonQ Aria-1 debiasing data

We sampled 5000 shots on IonQ's Aria-1 quantum computer. Experimental results using IonQ's debiasing mitigation alone is shown for local density $n_j$ (Supplementary Figure 5), two-point correlation function $C_{ij}$ (Supplementary Figure 6), and site-averaged correlation $C(d)$ (Supplementary Figure 7). While all experimental data follows the general qualitative trend of the ED benchmark, we still see large deviations due to hardware noise.

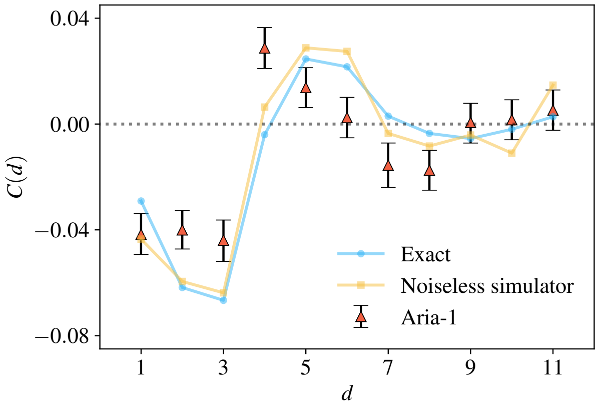


Supplementary Figure 4. Site-averaged correlation $C(d)$ over sites separated by $d = |i - j|$. Experimental results obtained without error-mitigation effort are compared with noiseless simulation of the optimized HVA (orange square) and exact values from ED (blue circle). Error bars indicate 68% confidence intervals obtained by means of percentile bootstrap.

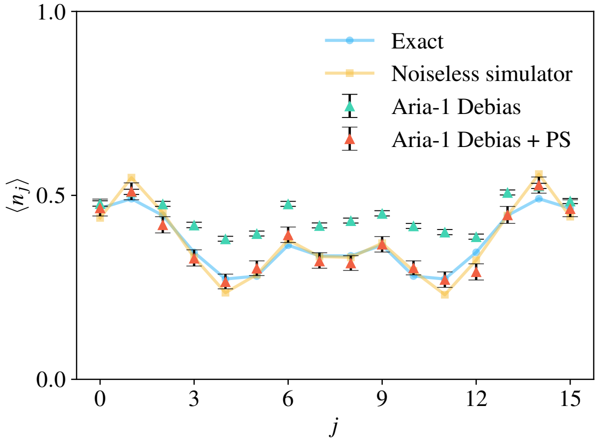


Supplementary Figure 5. Local density $n_j$ at site $j$ for system size $N_e = 6$. Experimental results with debiasing error mitigation (cyan triangle) and symmetry-verification postselection (red triangle) are compared with noiseless simulation of the optimized HVA (orange square) and exact values from ED (blue circle). Error bars indicate 68% confidence intervals obtained by means of percentile bootstrap.

### C. Symmetry-verification post-selection

To further mitigate errors arising from quantum hardware execution, we employ a symmetry-verification post-selection scheme. This method discards measurement bitstrings that violate the conservation of particle number, $\hat{N} = \sum_j \hat{n}_j$, and center-of-mass position, $\hat{K} = \sum_j j\hat{n}_j \pmod{N_\Phi}$. Specifically, only measurement bitstrings satisfying the following conditions are retained

$$N = 6, \; K = 13. \tag{15}$$

Supplementary Figure 8 and Supplementary Figure 9 show the measurement bitstring distributions for particle number and center-of-mass position, respectively. After debiasing, 24.9% of the measurements satisfy particle number conservation, while 14.6% satisfy center-of-mass conservation. Enforcing both symmetries yields a final selection rate of 10.4%.

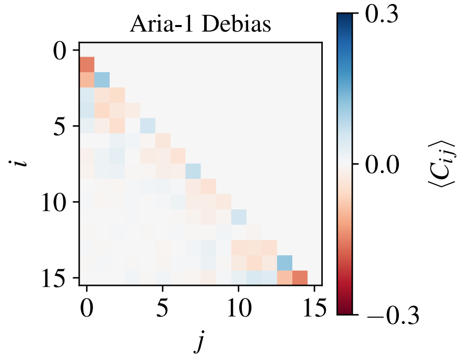


Supplementary Figure 6. Two-point correlation function $C_{ij}$ between site $i$ and $j$ with debiasing error-mitigation alone. We set $C_{ij} = 0$ for $i \leq j$ for clarity.

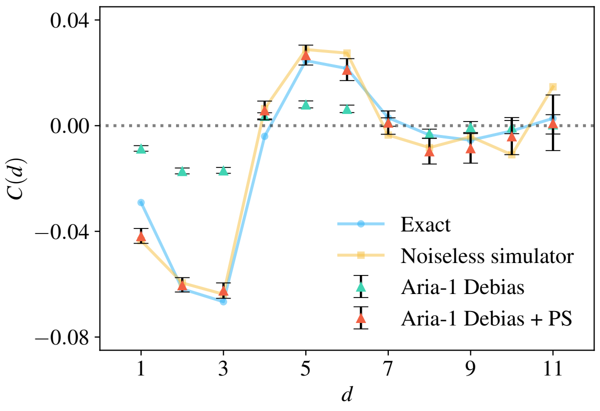


Supplementary Figure 7. Site-averaged correlation $C(d)$ over sites separated by $d = |i - j|$. Experimental results with debiasing error mitigation (cyan triangle) and symmetry-verification postselection (red triangle) are compared with noiseless simulation of the optimized HVA (orange square) and exact values from ED (blue circle). Error bars indicate 68% confidence intervals obtained by means of percentile bootstrap.

### D. IonQ Forte-1 extended data of second Rényi entropy measurement

To suppress edge effects from the open boundaries during the extraction of second Rényi entropy $S_A^{(2)}$, we choose three contiguous subsystem blocks of fixed size $N_A = 6$ centered around the middle of the cylinder, in the thin-torus root-pattern picture for $\nu = 1/3$,

$$A_1 = 1001|001001|001001, \tag{16}$$

$$A_2 = 10010|010010|01001, \tag{17}$$

$$A_3 = 100100|100100|1001, \tag{18}$$

where vertical bars indicate the subsystem blocks. These three partitions are separated from both open ends by at least four orbitals and lie around the density plateau region observed in the main text, ensuring that the extracted entanglement is dominated by bulk physics rather than boundary reconstruction. The resulting $S_A^{(2)}(L_y)$ data for each partition $A_1, A_2, A_3$ are shown in Supplementary Figure 10.

For finite cylinders in the Landau-gauge orbital representation, short-distance physics at the subsystem partition boundaries can produce a weak position dependence in the nonuniversal part of $S_A^{(2)}$. Previous work [3] explicitly demonstrates that, at fixed subsystem size, translated subsystem partitions can yield different entropy values, precisely because the microscopic boundary configuration controls the entanglement spectrum near the cut. They further employ an arithmetic average over translated partitions to suppress this oscillatory cut dependence and obtain a smoother scaling behavior.

To suppress this cut-dependent oscillations and improve statistical efficiency, we adopt the same arithmetic average

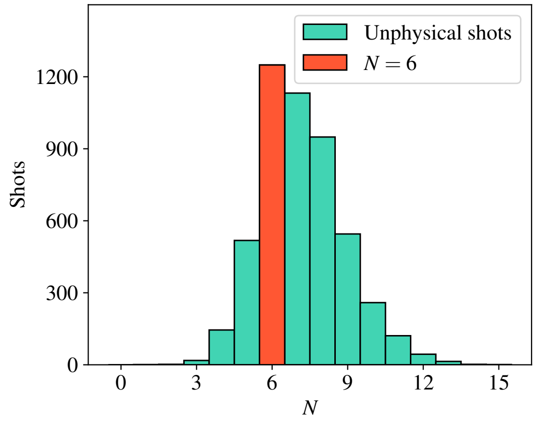


Supplementary Figure 8. Distribution of measurement bitstrings by particle number $N$. 24.9% of total shots (red) satisfy the particle number conservation ($N = 6$), while the remaining measurement bitstrings (cyan) are deemed unphysical and discarded.

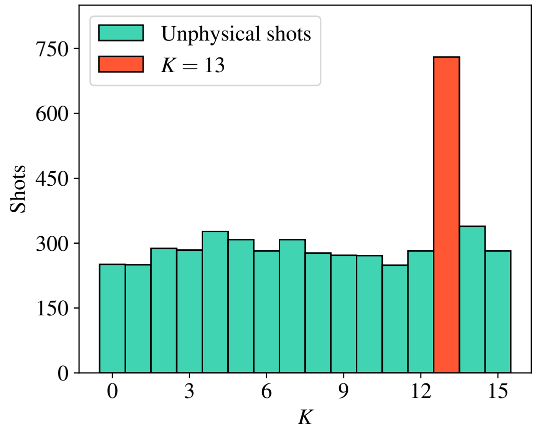


Supplementary Figure 9. Distribution of measurement bitstrings by center-of-mass position. Only 14.6% of total shots (red) satisfy the center-of-mass conservation ($K = 13$), while the remaining measurement bitstrings (cyan) are deemed unphysical and discarded.

over the three types of subsystem partition $A_1, A_2, A_3$. In practice, we compute $S_A^{(2)}(L_y)$ for each of the three subsystem partitions and report the entropy obtained with means of bootstrap.

[1] V. Crépel, N. Claussen, N. Regnault, and B. Estienne, Microscopic study of the halperin–laughlin interface through matrix product states, Nature communications **10**, 1860 (2019).
[2] T. Brydges, A. Elben, P. Jurcevic, B. Vermersch, C. Maier, B. P. Lanyon, P. Zoller, R. Blatt, and C. F. Roos, Probing Rényi entanglement entropy via randomized measurements, Science **364**, 260 (2019).
[3] A. M. Läuchli, E. J. Bergholtz, and M. Haque, Entanglement scaling of fractional quantum hall states through geometric deformations, New Journal of Physics **12**, 075004 (2010).

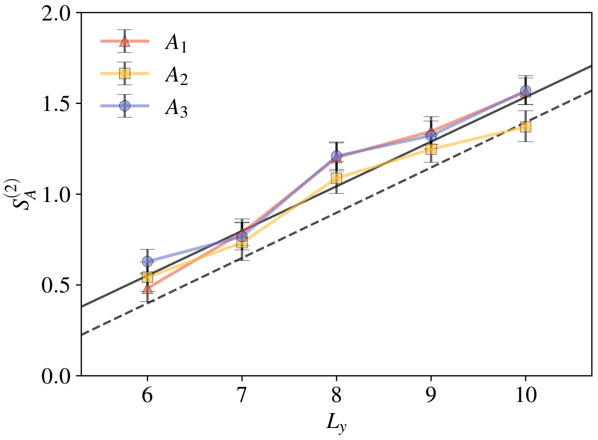


Supplementary Figure 10. Second Rényi entropies for subsystem partitions $A_1, A_2, A_3$ measured on Forte-1. Error bars denote 68% confidence intervals obtained by bootstrap. Colored lines are guide to eye. Area law fitted against Forte-1 measurements (black solid line) with $\alpha_{\text{exp}} = 0.245 \pm 0.021$ and $-\gamma_{\text{exp}} = -0.92 \pm 0.17$ and area law fitted against noiseless simulation (black dash line) with $\alpha_{\text{HVA}} = 0.249$ and $-\gamma_{\text{HVA}} = -1.09$ are shown for reference.

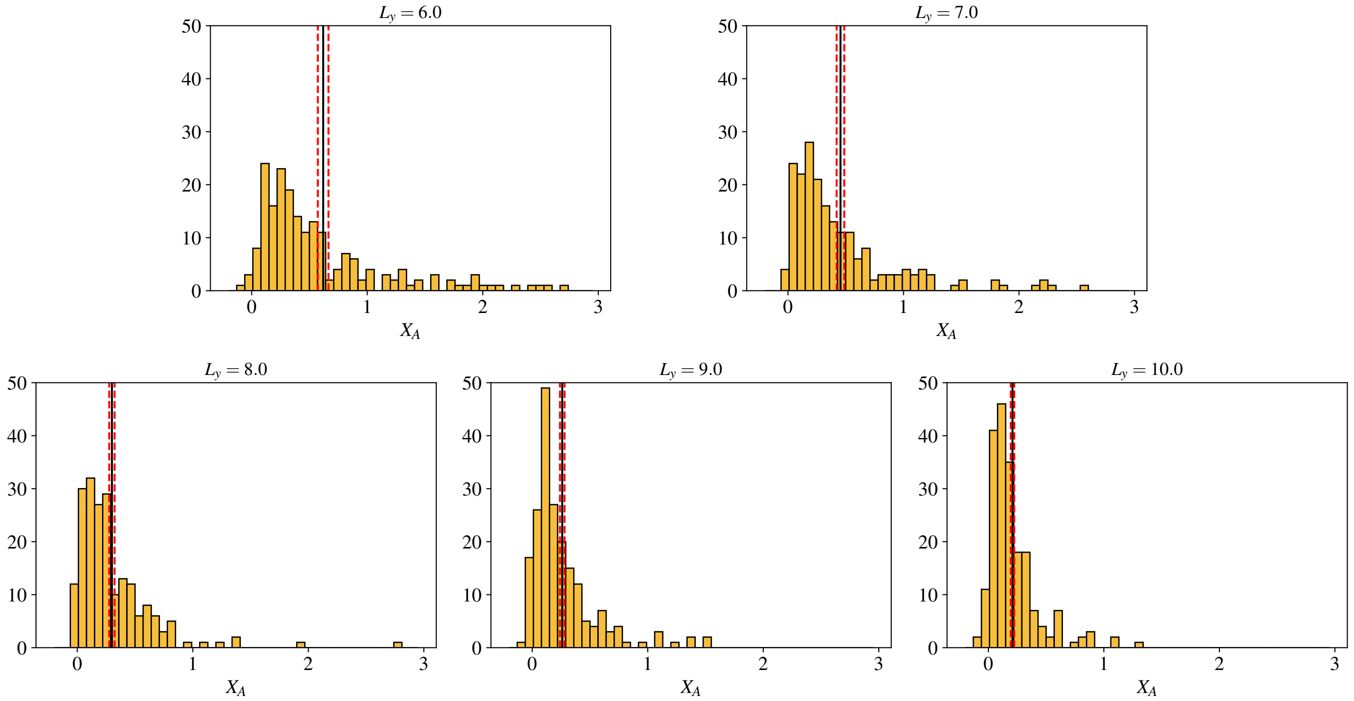


Supplementary Figure 11. Distribution of purity estimators from randomized measurements. Histograms display the distribution of the purity estimator $X_A$ for the six-qubit bulk subsystem $A_1$ across the ensemble of $N_U = 200$ random unitaries for cylinder circumferences $L_y \in \{6, 7, 8, 9, 10\}$. Black line indicates the mean purity value obtained via bootstrapping, while the red dashed lines denote the 68% confidence interval derived from percentile bootstrapping.

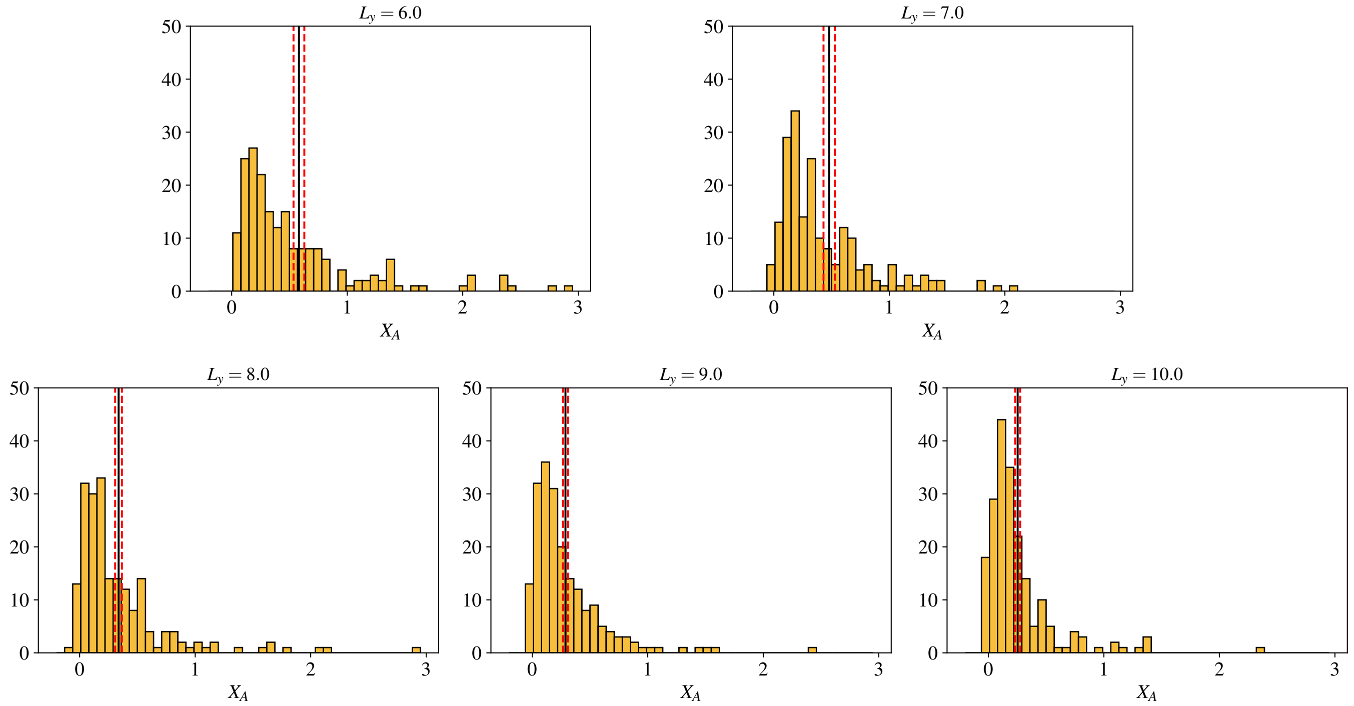


Supplementary Figure 12. Distribution of purity estimators from randomized measurements. Histograms display the distribution of the purity estimator $X_A$ for the six-qubit bulk subsystem $A_2$ across the ensemble of $N_U = 200$ random unitaries for cylinder circumferences $L_y \in \{6, 7, 8, 9, 10\}$. Black line indicates the mean purity value obtained via bootstrapping, while the red dashed lines denote the 68% confidence interval derived from percentile bootstrapping.

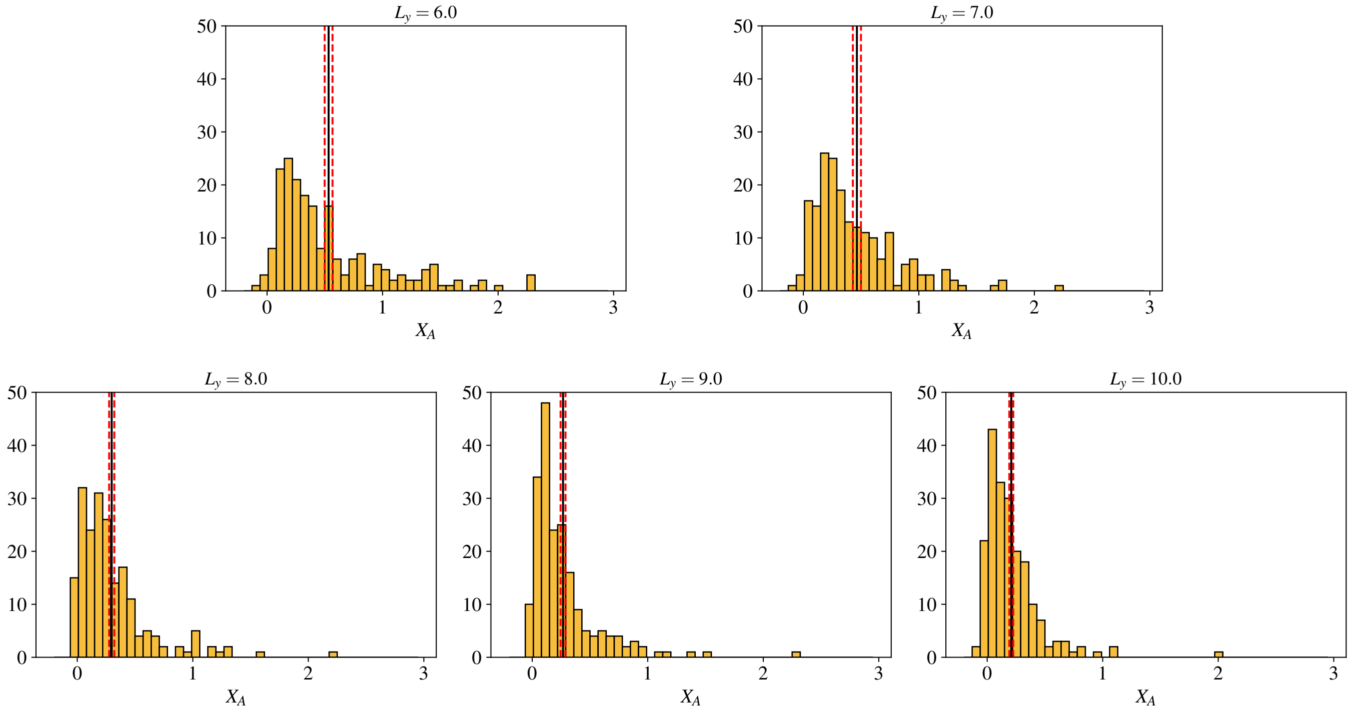


Supplementary Figure 13. Distribution of purity estimators from randomized measurements. Histograms display the distribution of the purity estimator $X_A$ for the six-qubit bulk subsystem $A_3$ across the ensemble of $N_U = 200$ random unitaries for cylinder circumferences $L_y \in \{6, 7, 8, 9, 10\}$. Black line indicates the mean purity value obtained via bootstrapping, while the red dashed lines denote the 68% confidence interval derived from percentile bootstrapping.
