# Supplementary material for: Realization of fermionic Laughlin state on a quantum processor
Source: Nat Commun. 2026 Jun 8;17:4919. doi: 10.1038/s41467-026-72769-y (PMC13247042; doi:10.1038/s41467-026-72769-y)
Supplement: Supplementary file 2 — Transparent Peer Review file [file 41467_2026_72769_MOESM2_ESM.pdf]

nature portfolio

Peer Review File

Realization of fermionic Laughlin state on a quantum processor

Corresponding Author: Professor Ting Cao

This file contains all reviewer reports in order by version, followed by all author rebuttals in order by version.

Version 0:

Reviewer comments:

Reviewer #1

(Remarks to the Author)
The paper “Realization of fermionic Laughlin state on a quantum processor” claims to report the first realization of the v = 1/3 fermionic Laughlin state on a quantum processor, specifically IonQ’s Aria-1 trapped-ion quantum computer. The authors develop a scalable, symmetry-preserving Hamiltonian variational ansatz (HVA) with 369 two-qubit gates on 16 qubits to prepare the Laughlin state efficiently. They claim to extract topological features like the correlation hole and bulk-edge correspondence with strong agreement to exact diagonalization benchmarks, employing symmetry-verification error mitigation to handle hardware noise.

In summary, I believe that very little new things have been learnt from this study. We already knew that removing terms from a Hamiltonian changes the overlap with the exact ground state. We already knew that we can use symmetry filtering of states to mitigate errors. We already knew that we can use small digital quantum computers to do stuff we knew would work because we can simulate everything with exact diagonalization. In addition, very little attempt has been made to explain the physical concepts probed and it is unclear how their approach will scale in the quantum advantage regime, which is super challenging for Laughlin states because of the closing gap.

The data accumulated in this study could be used to answer some genuinely interesting questions. For example, how is it possible that the agreement with the exact results are so good despite the ridiculously bad 2Q gate fidelity? Is there some reason why the properties probed should be more resilient to errors?

More comments:
- It is unclear what the difficulties were that were overcome. Why has fermionic Laughlin state not been prepared yet? What is difficulty compared to bosonic topologically ordered states?
- It is also unclear what the novelty of their work is. Presumably many people have before studied the effects of truncating the interaction?
- It is unclear how scalable their approach is. In fact, the statements about scalability are way too strong. They studied extremely small systems in the range of exact diagonalization, from which no sensible statements about scalability can be made.
- What exactly is the truncation criterion? Fidliety F=x%? The discussion below Eq.(2) makes it sound as if V31 was sort of put in by hand.
- It is not studied how the circuit depth or gate count scales with the truncation
- Of course it's nice to lower the number of variational parameters as discussed in L214 (by the way, what does generalize mean here? Make uniform?), but is that valid, i.e. is the circuit still expressive enough to capture the ground state for large systems? The data in Fig. 3 is inconclusive - these are very small system sizes. What is needed is some analytical argument why this is sensible.
- Generally, the data is massively overinterpreted, leading to statements which directly contradict each other: In paragraph L315, the authors first state "leading to oscillatory behaviour" and then "away from boundaries there is a uniform plateau" -- how can this simultaneously be true? How can we see from the data that the edge states are chiral?
- Key literature on state preparation of other fermionic models has not been cited:
- Stanisic, ..., Montanaro Nat Comm 2022
- Hemery, .., Nigmatullin, PRX Quantum 2024
- Nigmatullin, ..., Dreyer, arXiv 2024

- Evered, ..., Lukin arXiv 2025
Many many missing concepts which makes understanding the manuscript all but impossible for people outside of the field of Laughlin states:
- What is |c_j| is line 127?
- What is ED in line 129?
- What is "interaction range"?
- What is N_e in Fig.1?
- Fig 1: What is magnetic length? Why are "landau level orbitals" shown in Fig. 1 and what are they? What is their relation to Eq.(1)?
- Why is hilbert space fragmentation mentioned? What is Krylov subspace?
Small comments:
- Typo in caption: "axiel"

Reviewer #2

(Remarks to the Author)
The authors present a method by which the fermionic Laughlin state can be realized efficiently on a quantum processor through finite range unitary operations. To achieve this, they employ an effective Hamiltonian obtained by mapping the interacting lowest Landau level to a one-dimensional interacting model and truncating the interaction, limiting the number of variational parameters. This effective Hamiltonian is used to build a unitary circuit that the authors claim results in the 1/3 fermionic Laughlin state with the depth of the circuit scaling linearly with system size. They provide evidence that they have realized the Laughlin state on the quantum processor by comparing the density and two-point correlation function with classical simulations and exact results from the full Hamiltonian. They also demonstrate that the realized state has a topological entanglement entropy consistent with the Laughlin state.

This work provides a promising method for realizing highly entangled states on quantum processors in a scalable and controllable way. This may be used to advance our understanding and ability to control strongly correlated topological phases. Additionally, the use of symmetry-verification error correction significantly improves the accuracy of their results.

Still there is one major reservation about the work presented, chiefly with the claim that they have verified the topological nature of the realized state. While the authors claim they observe the bulk-boundary correspondence, they only present the ground state density, claiming the accumulation on the edge is evidence of the chiral edge mode. This seems inaccurate, particularly because they should be realizing the ground state Laughlin wave function and thus the density does not provide a probe of the edge states directly. Besides, the result does not show that the edge modes (if any) are chiral, and no evidence of topological nature of the realized state is given. The most convincing evidence of the topological nature of the state provided is the topological entanglement entropy, which is actually obtained via the classical calculation and so not directly observed on from the quantum computation. Also looking at the fit, it is apparent there are strong finite size effects present, obscuring the result.

The article in its current form does not seem suitable for publication in Nature communications for this reason. It may be suitable if the authors can correct or better justify their use of the density as a signature of the underlying topology and provide stronger evidence of the topological nature of the state, for example, by demonstrating fractional charge pumping in the realized state.

Some smaller issues
1. In Fig. 1, I assume L_x is 10, but this should be stated explicitly.
2. |c_j| at the bottom of page 2 seems like an error. Should this be |V_{km}|?

Reviewer #3

(Remarks to the Author)
I co-reviewed this manuscript with one of the reviewers who provided the listed reports. This is part of the Nature Communications initiative to facilitate training in peer review and to provide appropriate recognition for Early Career Researchers who co-review manuscripts.

Reviewer #4

(Remarks to the Author)
The paper “Realization of fermionic Laughlin state on a quantum processor” has a lot of interesting research ideas, but it is not clear the ideas in the paper are important. It should certainly be published, somewhere. However, without any clear goals or realistic discussion of how the methods discussed would be able to advance the field of fractional quantum hall, it will have limited reach in its current form. However, I do want to mention a few good aspects of the paper.

1) The hardware results look great.
2) The writing is excellent (mostly), which is to say, the paper is easy to read even for non-experts.

Overview:

The papers attempt to carry over classical attempts to simulate fractional quantum hall on quantum hardware, but without solving any of the actual problem on quantum hardware. Everything is determined on classical hardware, including the variational parameters of the circuits. The actual research performed on quantum hardware is mostly that of tomography and therefore a test of the hardware. While I do consider these ideas important to test, the methodology in the paper does not present a clear path to making new predictions in the field of fractional quantum hall physics. The methodology in the paper is not particularly new, although it is a nice result to show that the HVA ansatz can produce Laughlin wavefunctions with high fidelity.

To summarize the main research ideas explored in the paper:
1) The authors are solving the parent Hamiltonian of a known state, and even then, the parent Hamiltonian is too hard to solve on quantum hardware, they have to approximate it further. The number of second quantized terms in the parent Hamiltonian is less than 10 in the current manuscript.
2) The ansatz under consideration is somewhat trial to the extent that it can be simulated with five variational parameters, which is perhaps too trivial. See the next comment.
3) It is not clear is how the authors define success. Matching a known wave function for an extremely truncated Hamiltonian is not the same as predicting the ground state of a fractional quantum hall state. The authors present fidelity numbers throughout the paper, but at no point do the authors determine what is a minimal number for success, or even what a minimal fidelity number would be for utility/advantage. I do realize that there is some discussion of this on line 170, but that not extensive enough.
4) The scaling arguments in the paper are not robust. It is clear from figure 1) that errors will grow with increasing system size. Even more so: six to eight electrons is not enough data to extrapolate. System sizes up to 11 sites is not convincing either. Table 1 shows impressive numbers or two qubit gates, but the ansatz does not entangle all qubits when the system sizes get large, which casts doubts on its viability, or the difficulty of the actual problem.

Issues with regards to the ansatz:
The authors are proposing a 5-parameter ansatz for all system sizes. However, that will necessarily create a finite light cone of entanglement. The wave function ansatz will not be able to realize arbitrary long-range entanglement. Even more so, it is likely such ansatz with limited entanglement in many cases can be solved efficiently (or sampled efficiently) on classical hardware.

From a big picture perspective I would ask he authors to consider what success would be and how to make advances in the field of fractional quantum hall. It is impossible to determine that from the current manuscript.

Other things to consider:
1) Line 154: The fidelity naturally decays with Ne due to the orthogonality catastrophe of many-body systems [28, 29].
I don’t consider this a useful statement. Whether or not the orthogonality catastrophe is important, the authors are using small system sizes with fixed number of variational parameters. The idea of the orthogonality catastrophe is not important for this situation at these system sizes.
2) Line 245: Optimizing |ψ({βj})⟩ eff with larger system size did not achieve higher fidelity (see Supplementary Information), indicating that the accumulation of infidelity is mainly due to the orthogonality catastrophe [28, 29].
I don’t think I even understand this statement. What does the orthogonality catastrophe have to do with this observation.
3) Line 421: In addition, our work introduces a new quantum methodology for studying strongly correlated topological materials.
My understanding of this work is that you are using an HVA like ansatz which is not even being optimized on quantum hardware. What is the “new quantum methodology”?
4) Line 160: the effective Hamiltonian HTT forms a Krylov subspace K, an example of Hilbert space fragmentation [30], which can be used to map FQH model under TT
I didn’t understand this point (or the rest of this paragraph). A Hamiltonian does not form a Kyrlov subspace as far as I know. The application of a Hamiltonian to a wavefunction multiple times can create a Kyrlov subspace. Could the authors expand or improve the writing in this paragraph?

Version 1:

Reviewer comments:

Reviewer #1

(Remarks to the Author)
The authors have made a genuine effort addressing my (admittedly quite demanding and critical) comments. I think among all of the studies that prepare states on current quantum computing hardware which are classically solvable, this is among the best-executed. In particular, the determination of the top. entanglement entropy is an impressive demonstration.

I still have reservations about the scalability of this approach. VQE is intrinsically very challenging to scale. However, in the present study, the authors present several noteworthy techniques to reduce the overhead which make it not impossible to think that this specific approach may be more scalable than usual VQE studies. I do agree with the author's rebuttal of my comments in that a combination of all of these existing techniques is actually a nontrivial feat.

Reviewer #2

(Remarks to the Author)
After reading the revised manuscript and replies to the reports, I have concluded this work is suitable for publication in Nature Communications. The authors have substantially improved the clarity of the work and its novelty through their rewrites as well as its impact by including additional calculations to confirm the nature of the state.

There is only one point I would request be clarified throughout the text. I believe when the authors refer to the exact and Laughlin state, they mean the same state obtained via exact diagonalization. This mixing of terminology is a bit confusing as sometimes it makes one think they are using the trial wave function. I recommend that the authors refer to this only as the exact state throughout and mention briefly upon introducing the state that it is known to match very well with the Laughlin trial wave function.

Reviewer #3

(Remarks to the Author)
I co-reviewed this manuscript with one of the reviewers who provided the listed reports. This is part of the Nature Communications initiative to facilitate training in peer review and to provide appropriate recognition for Early Career Researchers who co-review manuscripts.

Open Access This Peer Review File is licensed under a Creative Commons Attribution 4.0 International License, which permits use, sharing, adaptation, distribution and reproduction in any medium or format, as long as you give appropriate credit to the original author(s) and the source, provide a link to the Creative Commons license, and indicate if changes were made.
In cases where reviewers are anonymous, credit should be given to 'Anonymous Referee' and the source.
The images or other third party material in this Peer Review File are included in the article's Creative Commons license, unless indicated otherwise in a credit line to the material. If material is not included in the article's Creative Commons license and your intended use is not permitted by statutory regulation or exceeds the permitted use, you will need to obtain permission directly from the copyright holder.
To view a copy of this license, visit https://creativecommons.org/licenses/by/4.0/

Referee #1

Referee's comment #1:

The paper "Realization of fermionic Laughlin state on a quantum processor" claims to report the first realization of the $\nu = 1/3$ fermionic Laughlin state on a quantum processor, specifically IonQ's Aria-1 trapped-ion quantum computer. The authors develop a scalable, symmetry-preserving Hamiltonian variational ansatz (HVA) with 369 two-qubit gates on 16 qubits to prepare the Laughlin state efficiently. They claim to extract topological features like the correlation hole and bulk-edge correspondence with strong agreement to exact diagonalization benchmarks, employing symmetry-verification error mitigation to handle hardware noise.

Authors' reply: We thank the referee for their thorough evaluation of our manuscript. We will address the general and specific comments below.

Referee's comment #2:

In summary, I believe that very little new things have been learnt from this study. We already knew that removing terms from a Hamiltonian changes the overlap with the exact ground state. We already knew that we can use symmetry filtering of states to mitigate errors. We already knew that we can use small digital quantum computers to do stuff we knew would work because we can simulate everything with exact diagonalization. In addition, very little attempt has been made to explain the physical concepts probed and it is unclear how their approach will scale in the quantum advantage regime, which is super challenging for Laughlin states because of the closing gap.

Authors' reply:

We respectfully disagree with this assessment. While each individual ingredient (interaction truncation, symmetry-verification error-mitigation, small-scale digital quantum computer experiment with classical simulator verification) is known, our work is, to the best of our knowledge, the first concrete end-to-end demonstration on commercial trapped-ion hardware that these ingredients are integrated to prepare a fermionic $\nu = 1/3$ Laughlin state and to extract multiple, hallmark topological signatures directly from experimental data.

Ultimately, what is new here is how we combine these ingredients to simulate a realistic material-intrinsic electronic Hamiltonian on a digital quantum processor, in contrast to prior exactly solvable stabilizer models such as the surface code. We introduced and applied explicit criteria to truncate this Hamiltonian and used the resulting effective Hamiltonian to construct a symmetry-preserving HVA for state preparation. The integration required for the successful application to the FQH problem is a non-trivial scientific and engineering challenge, and its successful execution represents a significant step forward in the simulation of topological quantum matter. We believe that this end-to-end demonstration clearly provides a concrete and useful guide for future researchers extending this line of work.

Regarding the physical concepts, we present our experimental data that directly probes and quantitatively captures the defining physical characteristics of the Laughlin state-namely the expected bulk-edge correspondence (Fig. 4), the correlation hole of the incompressible electron liquid (Fig. 5) and the topological entanglement entropy (Fig. 6). We will elaborate on scalability in our detailed responses below.

Additionally, we would like to clarify on referee's question about "closing gap" that the relevant object is the bulk gap. On a finite open cylinder, the global many-body spectrum is gapless because of chiral edge modes, but the bulk of the $\nu = 1/3$ Laughlin phase remains gapped and is expected to converge to a finite value as the system size increases. Recent generalized adiabatic theorem[1] for extended fermionic systems show that adiabatic preparation of bulk observables and states is governed by the bulk gap even if the finite samples host gapless edges. Since our protocol targets bulk physics, it does not intrinsically run into a "closing gap" obstruction as system size grows.

Referee's comment #3:

[1] J. Henheik and S. Teufel, Adiabatic theorem in the thermodynamic limit: Systems with a gap in the bulk, Forum of Mathematics, Sigma 10, e4 (2022).

The data accumulated in this study could be used to answer some genuinely interesting questions. For example, how is it possible that the agreement with the exact results are so good despite the ridiculously bad 2Q gate fidelity? Is there some reason why the properties probed should be more resilient to errors?

Authors' reply: We thank the referee for this insightful question, as it highlights a critical and effective aspect of our methodology. The high agreement between our hardware results and the exact benchmarks is not accidental but a direct consequence of our error mitigation strategy that is enabled by our symmetry-preserving nature of our Hamiltonian variational ansatz. On IonQ Aria-1, debiasing mitigation[2] suppresses systematic errors such as coherent over-rotations and reduces hardware-induced bias in expectation values. Secondly, our Hamiltonian-variational ansatz is symmetry-preserving by construction; our symmetry-verification post-selection project results back into the correct particle number and center-of-mass position sector, allowing us to mitigate symmetry-breaking component in the hardware noise.

Additionally our primary observables, local densities n_j (Pauli weight-1) and two-point correlation function C_{ij} (Pauli weight-2) are both low Pauli weight observables. Under common noise models (e.g., Pauli/depolarizing channels), the bias in the expectation of a Pauli string scales with its weight, so low-weight observables retain better signals even in the presence of noise.

To make this concrete, we include additional Aria-1 data without error-mitigation in Supplementary Information under section "Quantum hardware data" and demonstrate gradual improvement in n_j and C_{ij} through each stage of error mitigation.

Referee's comment #4:

More comments:

- It is unclear what the difficulties were that were overcome. Why has the fermionic Laughlin state not been prepared yet? What is the difficulty compared to bosonic topologically ordered states?
- Authors' reply: We appreciate the referee's question and the opportunity to clarify this point. In our view, preparing a fermionic Laughlin state on a digital quantum processor is substantially more demanding than the bosonic topologically ordered states and stabilizer codes that have been realized so far. Existing demonstrations of topological order on quantum hardware have predominantly focused on stabilizer-type spin models such as the surface code, whose Hamiltonians are sums of local, commuting Pauli terms and whose ground states could admit relatively shallow and near-optimal preparation circuits [3] [4]. These models do not require simulating a realistic electronic Hamiltonian.

  By contrast, the fermionic Laughlin state is a highly entangled, non-stabilizer wavefunction: it is the ground state of a strongly interacting Landau level Hamiltonian that lacks simple mapping to state preparation circuits. A naive implementation of VQE or HVA to such material-realistic Hamiltonian leads to circuit consuming resources that far exceed current hardware capabilities. In parallel, bosonic Laughlin analogs have been realized primarily in analog simulators, such as rotating Bose gases [5] and photonic lattice [6], where the experimental hardware is specifically engineered to reproduce the effective interactions. As a result, the insights gained there do not straightforwardly transfer to the broader goal of realizing topological order on universal digital quantum processors.

  Our experiment overcomes precisely this set of challenges in preparing a genuine fermionic Laughlin

[2] A. Maksymov, J. Nguyen, Y. Nam, and I. Markov, Enhancing quantum computer performance via symmetrization (2023), arXiv:2301.07233

[3] Kitaev, A. Yu. "Fault-tolerant quantum computation by anyons." Annals of physics 303.1 (2003): 2-30.

[4] Tantivasadakarn, N., Verresen, R., Vishwanath, A. (2023). Shortest route to non-Abelian topological order on a quantum processor. Physical Review Letters, 131(6), 060405.

[5] Gemelke, N., Sarajlic, E., Chu, S. (2010). Rotating few-body atomic systems in the fractional quantum Hall regime. arXiv preprint arXiv:1007.2677.

[6] Wang, C., Liu, F. M., Chen, M. C., Chen, H., Zhao, X. H., Ying, C., ..., Pan, J. W. (2024). Realization of fractional quantum Hall state with interacting photons. Science, 384(6695), 579-584.

state on a digital quantum processor. We believe these difficulties are already highlighted in the second paragraph of introduction.

- It is also unclear what the novelty of their work is. Presumably, many people have studied the effects of truncating the interaction before?
- Authors' reply: We would like to clarify that our work's novelty regarding constructing the effective Hamiltonian is not in the act of truncation itself but lies instead in our dual-criteria methodology for constructing an effective Hamiltonian for the HVA. Previous works studying effects of truncation only focus on phase diagrams and the ground state properties. They do not aim to engineer an effective Hamiltonian variational ansatz. Our contribution is to turn truncation into a design principle for quantum circuits.
- It is unclear how scalable their approach is. In fact, the statements about scalability are way too strong. They studied extremely small systems in the range of exact diagonalization, from which no sensible statements about scalability can be made.
- Authors' reply: We appreciate the referee for raising this concern and we have performed major revision to the section "Quantum Circuit for state preparation" to incorporate circuit's gate/depth scaling analysis in our main text to clarify our scalability statements:

  > "We interpret the HVA as a digitized adiabatic protocol generated by a local effective Hamiltonian. Lieb–Robinson bounds on the spread of correlations under local dynamics imply an effective light cone with finite velocity. We therefore expect that, for our Laughlin state HVA, the number of repetitions p must grow at least linearly with the system size in order to faithfully reproduce the long-range entanglement structure of the topological phase. As we show below, our ansatz also achieves a linear scaling of total number of variational parameters by generalizing parameters in a HVA layer across the lattice. This avoids the quadratic or worse parameter growth that would result from assigning independent parameters to every microscopic term and aligns with previous work showing that constrained HVA remains expressive while improving trainability."
  >
  > "As a result, each HVA repetition uses five independent parameters, independent of the system size. This dimensionality reduction of parameter space not only simplifies the variational optimization but also ensures the total number of parameters grows only through the number of HVA repetitions p, i.e., $N_{\text{param}} \sim \mathcal{O}(p) \propto N_e$."

  As we clarified in the revised main text, the number of repetition p of our geometrically local Hamiltonian variational ansatz needs to grow linearly in system size as imposed by the Lieb-Robinson bound. This matches tight results for synthetic topological orders, where any local unitary encoder needs circuit depth scale linearly with system size such as the case for surface code[7] and it matches our interpretive stance that HVA is a digitized compressed adiabatic protocol whose runtime maps to depth. Our comparison to system sizes accessible by exact diagonalization (shown in Fig. 3 in main text) indicate that, this HVA constructed from effective Hamiltonian already produces observables such as local densities and correlation functions with good accuracy, suggesting that the prefactor required for circuit depth scaling v.s. system size can be modest and motivating near-term extensions as hardware quality continues to improve. Our reported sizes (accessible through exact diagonalization) were chosen to validate the physics rather than to claim asymptotic advantage. We now clarified explicitly in the main text that our experiments are near-term demonstration and validation of the integrated pipeline.
- What exactly is the truncation criterion? Fidelity $F = x\%$? The discussion below Eq. (2) makes it sound as if V_{31} was sort of put in by hand.
- Authors' reply: The truncation is not fixed by a single absolute fidelity threshold $F = x\%$ and it should not be. In many-body systems, global ground-state overlaps between two non-identical Hamiltonians

[7] O. Higgott, M. Wilson, J. Hefford, J. Dborin, F. Hanif, S. Burton, and D. E. Browne, Optimal local unitary encoding circuits for the surface code, Quantum 5, 517 (2021).

in the same phase will decrease with increasing system size, so a single value of F as an absolute criterion is not meaningful. As a result, in our case, the first, quantitative criterion works in a comparative sense. The obtained fidelity shows that $k+m \leq 3$ is too short-ranged, and that one must go to $k+m=4,5$ to reach fidelity $\mathcal{F} > 0.9$ with the Laughlin state at $N_e = 6$. Our second, qualitative criterion then reinforces this in the definitive sense: for $k+m \leq 3$ the ground state of H_{eff} is confined to a Krylov subspace and fails to reproduce the correct entanglement scaling, whereas $k+m \leq 4$ breaks this constraint and restores the expected entanglement area-law. The difference between $k+m \leq 3$ and $k+m \leq 4$ is precisely the inclusion of V_{40} and V_{31} interactions; among these, V_{31} plays the dominant role by providing off-diagonal scattering that alters the wavefunction structure, while V_{40} is purely diagonal and primarily shifts energies. We therefore make a physics-informed decision to keep the minimal set: retaining V_{31} but not V_{40} because the relevant "error budget" includes both algorithmic and hardware errors, and resource usage must be controlled even in fault-tolerant setting in the future. We have already explained this truncation strategy and its physical motivation in the following paragraph in the manuscript. We believe the criteria are stated clearly and we made minor revisions to improve readability.

> "We note that at the interaction range $k+m=4$, we retain only the off-diagonal scattering term V_{31} in H_{eff}, which plays a crucial role in shaping the wavefunction structure and avoiding Hilbert space fragmentation. In contrast, V_{40}, despite falling within the same interaction range, is a diagonal electrostatic term that primarily results in energy shifts without significantly influencing the wavefunction. To further reduce circuit depth, we exclude V_{40} from H_{eff}."

- It is not studied how the circuit depth or gate count scales with the truncation.
- Authors' reply: We added a section analyzing how the HVA circuit's CNOT gate and depth scales with the truncation and system size in the supplementary information Sec. III B. The CNOT gate count grows roughly linearly with system size and exponentially with the interaction range truncation while the CNOT depth scales approximately exponentially with the interaction range truncation and only weakly with system size.
- Of course it's nice to lower the number of variational parameters as discussed in line 214 (by the way, what does *generalize* mean here? Make uniform?), but is that valid, i.e., is the circuit still expressive enough to capture the ground state for large systems? The data in Fig. 3 is inconclusive — these are very small system sizes. What is needed is some analytical argument why this is sensible.
- Authors' reply: We thank the referee for this question. Our reduction in number of parameters follows the HVA design principle of using a small set of physically motivated generator families (in our case the $\hat{U}_{km}$'s) per repetition and tying parameters due to similarity in mathematical structures across the lattice sites j. Such problem-informed constraints are not only standard in HVA[8] but also improves trainability and mitigates barren plateau problem. By "generalize" we mean to use the same β_{km} parameter for the unitary $\hat{U}_{km}$ on different site j. This is a constrained HVA approach[9], where one varies one parameter per physical generator (in our case, one parameter per $\hat{U}_{km}$ layer), rather than one per microscopic term. We included the following sentence to the main text to clarify:

> "In practice, this means that all gates within the same unitary $\hat{U}_{km}$ share the same parameter β_{km}, yielding a constrained HVA with one variational parameter per physical generator $\hat{U}_{km}$ rather than one per microscopic term. As a result, each HVA repetition uses five independent parameters, independent of the system size."

[8] D. Wecker, M. B. Hastings, and M. Troyer, Progress towards practical quantum variational algorithms, Phys. Rev. A 92, 042303 (2015).

[9] C.-Y. Park and N. Killoran, Hamiltonian variational ansatz without barren plateaus, Quantum 8, 1239 (2024).

To clarify the confusion about scaling implication: tying parameters only reduces the number of parameters per HVA repetition. Expressivity at larger sizes is achieved not by adding per-term parameters, but by increasing the number of repetitions p. As we mentioned in responding previous concern: the total number of parameters will scale linearly with system size as HVA repetition needs to grow linearly in system size. We added this clarification as the following sentence to the main text:

> "As a result, each HVA repetition uses five independent parameters, independent of the system size. This dimensionality reduction of parameter space not only simplifies the variational optimization but also ensures the total number of parameters grows only through the number of HVA repetitions p, i.e., $N_{\text{param}} \sim \mathcal{O}(p) \propto N_e$."

In the main text, we position Fig. 3 as empirical evidence to support a transferability (warm-start) picture rather than to claim asymptotic expressivity from small ED sizes. Specifically, at fixed family set and fixed repetition p, we observe that parameters optimized at $N_e = 6$ remain effective when transferred to larger systems (up to $N_e = 10$) for local observables (density and two-point correlators), while re-optimizing at larger size within the same ansatz yields limited improvement in global fidelity. This behavior is consistent with recent results showing that "smooth" HVA solutions can transfer across system sizes and mitigate trainability barriers, and with theoretical analyses demonstrating that suitably constrained HVA constructions can avoid barren-plateau behavior under appropriate conditions[10]. We have revised the related section in main text to emphasize this conclusion:

> "Optimizing $|\psi(\{\beta_j\})\rangle_{\text{eff}}$ with larger system size did not achieve higher fidelity (see Supplementary Information), further supporting parameter transferability and our construction's resilience to barren plateau. This smooth transferability suggests that parameters optimized on smaller systems provide high-quality warm starts for larger systems, reducing classical optimization costs and mitigating trainability issues when one subsequently increases the HVA repetition p with system size."

> "Notably, the average deviation of intensive quantities, such as the local density and two-point correlation between $|\psi(\{\beta_j\})\rangle_{\text{eff}}$ and the Laughlin state, remain constant with increasing system size (Fig. 3(b-c)). This observation strengthens the smooth transferability and suggests that for H_{eff} considered here, reproducing local physics with high accuracy, does not require large prefactors in the linear depth scaling of the HVA."

- Generally, the data is massively overinterpreted, leading to statements which directly contradict each other: In paragraph L315, the authors first state "leading to oscillatory behaviour" and then "away from boundaries there is a uniform plateau" — how can this simultaneously be true? How can we see from the data that the edge states are chiral?
- Authors' reply: We respectfully disagree with the assessment that we stated contradictory statements and would like to point out that the referee has misinterpreted our data and statement. The "oscillatory behavior" we are referring to is the oscillation of local density n_j in sites near $j = 0$ and $j = 15$ shown in Fig. 4, as we have already stated in the main text

> "edge density structure is distinctly identified with an overdensity near the system boundaries ($j = 0, 15$) and subsequent oscillatory deviations of $\langle n_j \rangle$ from the bulk filling fraction $\nu = 1/3$."

The $j = 0$ and $j = 15$ sites represent the two physical open end of the cylinder geometry. Then it is

[10] A. A. Mele, G. B. Mbeng, G. E. Santoro, M. Collura, and P. Torta, Avoiding barren plateaus via transferability of smooth solutions in a Hamiltonian variational ansatz, Phys. Rev. A 106, L060401 (2022).

straightforward to understand "away from boundary there is a uniform plateau" refer to the region centered around site $j = 7, 8$.

We appreciate the referee's concern that local density we observed does not definitively prove the chirality of the state. We have revised the manuscript accordingly where density profile serves as a consistency check for the expected edge and bulk density structure from the Laughlin state. Such data is to be considered in conjunction with other diagnostics such as correlation and entanglement.

Referee's comment #5: Key literature on state preparation of other fermionic models has not been cited:

- Stanisic, *et al.*, Montanaro, Nat. Commun. 2022
- Hemery, *et al.*, Nigmatullin, PRX Quantum 2024
- Nigmatullin, *et al.*, Dreyer, arXiv 2024
- Evered, *et al.*, Lukin, arXiv 2025

Authors' reply: We appreciate the referee for pointing out the above literature, and we have included them in the revised main text.

Referee's comment #6:

Many missing concepts make understanding the manuscript nearly impossible for people outside of the field of Laughlin states:

- What is $|c_j|$ in line 127?
- What is ED in line 129?
- What is "interaction range"?
- What is N_e in Fig. 1?
- Fig. 1: What is magnetic length? Why are "Landau level orbitals" shown in Fig. 1 and what are they? What is their relation to Eq. (1)?
- Why is Hilbert space fragmentation mentioned? What is the Krylov subspace?
- Small comments:
  - Typo in caption: "axiel"

Authors' reply: We appreciate the referee for the careful read. We have corrected the typo mentioned. The symbol c_j on line 127 was a typo and we have corrected it to $|V_{km}|$.

We respectfully disagree with the evaluation that the manuscript is not accessible for the broader audience. We believe the following items are already defined at first use in the manuscript: "ED" in introduction and N_e (number of electrons) is clearly stated in the Fig. 1 caption. We have added the symbolic definition of"interaction range" in "The model" section at

> "This repulsive interaction decays at different rates for different interaction ranges $(k + m)$ as the cylinder's circumference L_y increases"

Additionally we expect the concept of magnetic length and Landau level orbitals to be common knowledge among broader physics community. We have also explained these concepts and their relationships to Fig. 1 and Eq. (1) in the Fractional quantum hall Hamiltonian section in Methods.

We believe the reason why Hilbert space fragmentation is mentioned is already explicit enough in main text. With additional discussion included in Supplementary Information Sec I.A. We have improved the writing regarding the Krylov subspaces in the main text to avoid any potential confusion:

> "the action of the effective Hamiltonian H_{TT} on the CDW state $|\Psi_{\mathrm{CDW}}\rangle$ forms a Krylov subspace

$\mathcal{K}(H_{\mathrm{TT}}, |\Psi_{\mathrm{CDW}}\rangle)$."

Thus, we believe the manuscript is self-contained at the level of customary for this journal.

Referee #2

Referee's comment #1:

The authors present a method by which the fermionic Laughlin state can be realized efficiently on a quantum processor through finite range unitary operations. To achieve this, they employ an effective Hamiltonian obtained by mapping the interacting lowest Landau level to a one-dimensional interacting model and truncating the interaction, limiting the number of variational parameters. This effective Hamiltonian is used to build a unitary circuit that the authors claim results in the 1/3 fermionic Laughlin state with the depth of the circuit scaling linearly with system size. They provide evidence that they have realized the Laughlin state on the quantum processor by comparing the density and two-point correlation function with classical simulations and exact results from the full Hamiltonian. They also demonstrate that the realized state has a topological entanglement entropy consistent with the Laughlin state.

Authors' reply: We thank the referee for taking the time for reviewing this paper.

Referee's comment #2:

This work provides a promising method for realizing highly entangled states on quantum processors in a scalable and controllable way. This may be used to advance our understanding and ability to control strongly correlated topological phases. Additionally, the use of symmetry-verification error correction significantly improves the accuracy of their results.

Authors' reply: We thank the referee for praising and acknowledging the importance of our work.

Referee's comment #3:

Still there is one major reservation about the work presented, chiefly with the claim that they have verified the topological nature of the realized state. While the authors claim they observe the bulk-boundary correspondence, they only present the ground state density, claiming the accumulation on the edge is evidence of the chiral edge mode. This seems inaccurate, particularly because they should be realizing the ground state Laughlin wave function and thus the density does not provide a probe of the edge states directly. Besides, the result does not show that the edge modes (if any) are chiral, and no evidence of topological nature of the realized state is given. The most convincing evidence of the topological nature of the state provided is the topological entanglement entropy, which is actually obtained via the classical calculation and so not directly observed on from the quantum computation. Also looking at the fit, it is apparent there are strong finite size effects present, obscuring the result.

Authors' reply: We thank the referee for this careful assessment and we agree with the referee's concern that ground state density profile is insufficient to prove the topological nature or chirality of the state. We have revised the manuscript accordingly to state that the density profile is a consistency check for the Laughlin state, rather than a standalone evidence.

We appreciate the referee's concern that topological entanglement entropy (TEE) obtained via classical calculation limits its weight as evidence. Motivated by this suggestion, we have introduced new experimental results measuring TEE on IonQ's Forte-1 quantum computer in our main text (new Fig. 6 and associated main-text paragraph reproduced below with details in Supplementary Information). Specifically, we now measure the second Rényi entropy $S_A^{(2)}$ of three different bulk orbital partitions using randomized measurement protocol [1] and extract γ_{topo} under geometric deformation of the cylinder circumference L_y.

> "To demonstrate entanglement behavior beyond pairwise correlation, we directly measured the topological entanglement entropy γ_{topo} of our prepared state via geometric deformation of the cylinder circumference L_y on the quantum processor. This quantity, which reflects the quantum dimension of anyonic excitations, serves as a robust diagnostic of topological order. We optimized the HVA ansatz $|\psi(\{\beta_j\})\rangle_{\text{eff}}$ for a range of $L_y \in [6, 10]$ near the isotropic geometry limit,

[1] Brydges, Tiff, et al. "Probing Rényi entanglement entropy via randomized measurements." Science 364.6437 (2019): 260-263.

and applied a randomized measurement protocol to estimate the second-order Rényi entropy $S_A^{(2)} = -\ln \mathrm{Tr}\, \rho_A^2$ for three different subsystem partition A in the bulk region. (see Supplementary Information for details)

In Fig. 6, the experimentally measured $S_A^{(2)}$ shows the expected area-law scaling $S_A^{(2)} = \alpha L_y - \gamma_{\text{topo}}$ with a systematic drift to higher entropy due to hardware noise when compared to noiseless simulator benchmark. Fitting the measured second-order Rényi entropy to the area-law scaling, we extracted $-\gamma_{\text{exp}} = -0.92 \pm 0.17$ (68% confidence interval by bootstrap resampling of finite-shot randomized measurement estimator, see Methods). For the ideal $\nu = 1/3$ Laughlin state, $-\gamma_{\text{topo}} = -\ln\sqrt{3}$ and because our system partition introduces two entanglement boundaries, the expected value is $-\gamma_{\text{topo}} = -2\ln\sqrt{3} \approx -1.10$. The consistent behavior in $S_A^{(2)}$ and γ_{topo} between our experiments and the theory provides compelling evidence of the topological order of the prepared $\nu = 1/3$ Laughlin state. Our pairwise correlation and entanglement entropy measurements demonstrate the ability to access microscopic structures that underlies topologically ordered states on a quantum processor."

Regarding finite-size effects, we agree that any extraction of γ_{topo} from finite systems must be interpreted with care. In our setting, we mitigate boundary effects by choosing three subsystem A_1, A_2, A_3 around the bulk region (around site $j = 8$) well separated from the physical edges. We extract γ_{topo} through averaging the $S_A^{(2)}$ over three different partitions to suppress finite-size and partition-dependent oscillations to obtain a smoother scaling behavior[2]. Such partition introduces two entanglement cuts, for which the $\nu = 1/3$ Laughlin state predicts $-\gamma = -2\ln\sqrt{3}$. Within the near-isotropic window $L_y \in [6, 10]$ and our experimental quantum resources to collect statistics for randomized measurement, the measured $S_A^{(2)}$ exhibits area-law scaling with an extracted intercept $-\gamma_{\text{exp}} = -0.92 \pm 0.17$, providing compelling evidence of Laughlin-type long-range entanglement in the prepared state.

Referee's comment #4:

The article in its current form does not seem suitable for publication in Nature communications for this reason. It may be suitable if the authors can correct or better justify their use of the density as a signature of the underlying topology and provide stronger evidence of the topological nature of the state, for example, by demonstrating fractional charge pumping in the realized state.

Authors' reply:

We thank the referee for this critical and insightful suggestion to strengthen the evidence for topological order beyond local density. In this major revision of manuscript, we now present a coherent set of complementary diagnostics: local density, spatial correlations, and entanglement with each addressing a distinct aspect of the Laughlin state.

1. **Density (electron droplet structure on cylinder)**: We measure $\langle n_j \rangle$ and observe an approximately uniform bulk density plateau near $\nu = 1/3$ together with boundary-localized structure near the physical edges, consistent with the expected edge density structure of a Laughlin droplet on a cylinder. This serves as a consistency check for correct droplet formation and for defining a bulk partition used in the entanglement analysis.
2. **Spatial correlations (beyond one-point functions)**: We also present two-point correlation data that exhibits the correlation-hole physics characteristic of strongly correlated FQH liquids, going beyond what can be inferred from the one-body density alone.
3. **Entanglement entropy (nonlocal topological diagnostic, measured on hardware)**: Most importantly, we now directly measure the second-order Rényi entropy on the quantum processor using randomized measurements, and extract the TEE. This quantity is the standard entanglement-based

[2] Läuchli, A. M., Bergholtz, E. J., Haque, M. (2010). Entanglement scaling of fractional quantum Hall states through geometric deformations. New Journal of Physics, 12(7), 075004.

diagnostic of topological order. It is sensitive to long-range entanglement that cannot be reproduced by matching local observables such as density alone, as we have shown in a control experiment in Supplementary Information Sec IV.A.

Importantly, taken together all three measurements, these diagnostics provide mutually reinforcing evidence that the prepared state realizes the expected physics of the $\nu = 1/3$ Laughlin state on the finite cylinder accessible to current quantum computer hardware.

Referee's comment #5:

Some smaller issues

- In Fig. 1, I assume L_x is 10, but this should be stated explicitly.
- $|c_j|$ at the bottom of page 2 seems like an error. Should this be $|V_{km}|$?

Authors' reply: We appreciate the referee for the careful read. The cylinder's height L_x is not 10 in Fig. 1 but determined by the number of flux quanta $N_\phi = L_x L_y/(2\pi)$. We have added this clarification to Fig. 1's caption. We also corrected the typo: $|c_j|$ to $|V_{km}|$.

Referee #3

Referee's comment #1:

I co-reviewed this manuscript with one of the reviewers who provided the listed reports. This is part of the Nature Communications initiative to facilitate training in peer review and to provide appropriate recognition for Early Career Researchers who co-review manuscripts.

Authors' reply: We appreciate this referee for putting in their time and effort to review our manuscript, and provide invaluable feedback.

Referee #4

Referee's comment #1:

The paper "Realization of fermionic Laughlin state on a quantum processor" has a lot of interesting research ideas, but it is not clear the ideas in the paper are important. It should certainly be published, somewhere. However, without any clear goals or realistic discussion of how the methods discussed would be able to advance the field of fractional quantum hall, it will have limited reach in its current form. However, I do want to mention a few good aspects of the paper.

1) The hardware results look great.

2) The writing is excellent (mostly), which is to say, the paper is easy to read even for non-experts.

Authors' reply: We would like to thank the referee for the thoughtful comments with positive feedback on our experimental results and the clarity of our writing. Below, we provide a point-by-point response to the referee's comments, clarifying our goals and the future research avenues our work directly enables.

Referee's comment #2:

Overview:

The papers attempt to carry over classical attempts to simulate fractional quantum hall on quantum hardware, but without solving any of the actual problem on quantum hardware. Everything is determined on classical hardware, including the variational parameters of the circuits. The actual research performed on quantum hardware is mostly that of tomography and therefore a test of the hardware. While I do consider these ideas important to test, the methodology in the paper does not present a clear path to making new predictions in the field of fractional quantum hall physics. The methodology in the paper is not particularly new, although it is a nice result to show that the HVA ansatz can produce Laughlin wavefunctions with high fidelity.

Authors' reply: We respectfully disagree with this assessment that our research is mostly a test of the hardware. At the current stage of hardware development, fully hardware-optimized VQE/HVA for a strongly correlated material-intrinsic Hamiltonians of the system size considered here remains unfeasible. The current largest size experiment by Stanisic, et al[1] considers the same system size of 16 qubits on Fermi-Hubbard model which contains significantly fewer terms in the Hamiltonian than ours.

Within this context, the first contribution of our work lies in the end-to-end demonstration of state-preparation workflow for a fermionic, non-stabilizer topological state. Current demonstrations of topological order on quantum processor have mainly focused on stabilizer-type systems where it may admit shallow or near-optimal state preparation circuits such as the surface code [2]. On the contrary, the fermionic Laughlin state is a highly entangled, non-stabilizer ground state of a strongly interacting Landau level Hamiltonian that cannot be easily compiled into quantum circuit. Applying simple VQE or HVA protocols to such models requires gate depths and resources that are unfeasible for current quantum hardware. To overcome this, we carefully engineered a symmetry-preserving HVA derived from a fractional quantum Hall Hamiltonian and extracted phase diagnostics on hardware.

Moreover, motivated by the referee's later question, "how do you define success?", we emphasize that we systemize and demonstrate on hardware a scalable, observable-centric benchmarking framework for fermionic FQH simulation, designed to remain meaningful beyond exact diagonalization. In this light, we view the present work as both an end-to-end demonstration and a necessary benchmark-and-validation step along a concrete path toward predictive studies of digital quantum simulation of fermionic Fractional Quantum Hall physics in regimes without classical ground truth. We have performed major revision in the main text to

[1] S. Stanisic, J. L. Bosse, F. M. Gambetta, R. A. Santos, W. Mruczkiewicz, T. E. O'Brien, E. Ostby, and A. Montanaro, Observing ground-state properties of the Fermi-Hubbard model using a scalable algorithm on a quantum computer, Nat Commun 13, 5743 (2022).

[2] O. Higgott, M. Wilson, J. Hefford, J. Dborin, F. Hanif, S. Burton, and D. E. Browne, Optimal local unitary encoding circuits for the surface code, Quantum 5, 517 (2021).

convey this idea (we also refer the referee to our response in Question (3) in comment #3 below)

> Introduction: "We verify the successful preparation by directly measuring, on the quantum processor, the characteristic microscopic and topological diagnostics of the Laughlin state, including bulk-edge density structure, correlation holes, and topological entanglement entropy, which show strong agreement with exact-diagonalization (ED) benchmarks.
> ......
> This suite of FQH-specific, observable-centric criteria provides a problem-tailored benchmark for future simulations in regimes without classical ground truth, enabling digital quantum processors to make genuinely predictive statements about competing strongly correlated phases and their emergent properties."
>
> Discussion: "We validated our experiment by extracting FQH phase-diagnostic observables from hardware and sets a benchmark for future experiments on larger, classically intractable systems."

As the referee notes, it is indeed important to test these ideas in practice, and it is a nontrivial result that our HVA workflow is validated on current publicly available commercial hardware by preparing Laughlin wavefunctions with high fidelity. While some of the individual pieces of this end-to-end demonstration are known, our work presents a synergistic integration of Hamiltonian design, ansatz construction, and hardware error-mitigation strategy. We have revised the introduction section discussing the importance of our work to reflect this point.

> "This work thus represents the first realization of a fermionic $\nu = 1/3$ Laughlin state on a digital quantum processor using an end-to-end workflow. Our synergistic integration approach of Hamiltonian design, ansatz construction, and error mitigation strategy establishes a concrete workflow for digital simulations of strongly correlated topological matter and opens a route to harnessing topological orders for both fundamental physics research and quantum-information applications."

Referee's comment #3:

To summarize the main research ideas explored in the paper:

1) The authors are solving the parent Hamiltonian of a known state, and even then, the parent Hamiltonian is too hard to solve on quantum hardware, they have to approximate it further. The number of second quantized terms in the parent Hamiltonian is less than 10 in the current manuscript.

Authors' reply: We appreciate the referee for their summaries of the main results of our work. We would like to point out an error in the comment: the number of second quantized fermionic terms in the parent Hamiltonian we considered ($N_e = 6$) is 624 with the number of terms being 92 in H_{eff}, including hermitian conjugate.

2) The ansatz under consideration is somewhat trial to the extent that it can be simulated with five variational parameters, which is perhaps too trivial. See the next comment.

Authors' reply: We respectfully disagree that the ansatz is "trivial" because it uses five variational parameters. In our implementation, "five parameters" refers to the number of parameters per HVA repetition for a fixed H_{eff}. Such construction stands as one of the initial motivations of HVA: a modest set of physically motivated generators per step and improve trainability through certain physical constraint such as tying parameters across the system.

We would also like to highlight that a small parameter count does not imply easy classical simulability: each repetition still compiles into many entangling operations across the system which can be classically hard to simulate. We also refer the referee to our response in comment #4.

3) It is not clear is how the authors define success. Matching a known wave function for an extremely truncated Hamiltonian is not the same as predicting the ground state of a fractional quantum hall state. The authors present fidelity numbers throughout the paper, but at no point do the authors determine what is a minimal number for success, or even what a minimal fidelity number would be for utility/advantage. I do realize that there is some discussion of this on line 170, but that not extensive enough.

Authors' reply: We thank the Referee for this important question, which prompted us to sharpen how success is defined and communicated in the revised manuscript. We address the Referee's concern along two aspects: (i) clarifying the role of fidelity in our work, and (ii) revising an explicit, physics-motivated definition of success.

(i) Fidelity is employed in our work as a comparative diagnostic for selecting the effective Hamiltonian H_{eff}, not as the primary success criterion for the quantum simulation itself. Specifically, in Fig. 1(b), fidelity serves to rank different truncation choices ($k + m \leq 3, 4, 5$) against one another and to justify our selection of $k + m \leq 4$. We have revised manuscript to clarify this point:

> "evaluate the fidelity $\mathcal{F}$, defined as the wavefunction overlap between the Laughlin state and the ground state of H_{eff} consisting of truncated interactions as a comparative diagnostic across truncation ranges, rather than as an absolute threshold."

The Hamiltonian truncation itself is a controlled effective-model reduction whose validity we verify both quantitatively, via comparative fidelity where exact diagonalization (ED) is available, and qualitatively, by confirming that H_{eff} preserves the area-law entanglement scaling and topological class of the full Hamiltonian. In other words, fidelity is part of the model-construction protocol, not part of the physics observation about the prepared state.

(ii) We adopt an observable-centric definition of success. We define successful state preparation as simultaneously satisfying the below independent, mutually-reinforcing criteria:

1. **Microscopic liquid structure (Figs. 4 and 5).** The prepared state must exhibit the defining spatial signatures of an FQH liquid: (a) the edge/bulk density structure in local occupations, and (b) the correlation hole in the pair correlation function. These diagnostics are "present vs absent" indicators of FQH liquid behavior, and our experiment reproduces their characteristic structure in agreement with exact diagonalization benchmarks.
2. **Universal topological constant (Fig. 6, new in revised manuscript).** We introduced new experimental result that extract the topological entanglement entropy (γ_{topo}) on the quantum processor, which is a universal quantity determined by the total quantum dimension of the topologically ordered phase. For the ($\nu = 1/3$) Laughlin phase, the expected constant is ($-\gamma = -\ln\sqrt{3}$); because our bipartition introduces two entanglement boundaries, the expected intercept is ($-2\ln\sqrt{3} \approx -1.10$). In the experiment, fitting the measured second-order Rényi entropies yields ($-\gamma_{\text{exp}} = -0.92 \pm 0.17$), consistent with the theoretical target within (1σ). Importantly, reproducing the correct TEE in conjunction with the microscopic diagnostics of the first criteria is highly constraining: together, they rule out trivial short-range-entangled explanations and provide compelling evidence that the prepared state resides in the correct topological phase.

We explicitly introduced this definition in the revised Introduction paragraph:

> We verify the successful preparation by directly measuring, on the quantum processor, the characteristic microscopic and topological diagnostics of the Laughlin state, including bulk-edge density structure, correlation holes, and topological entanglement entropy, which show strong agreement with exact-diagonalization (ED) benchmarks. We deem the preparation successful only when these independent diagnostics are simultaneously satisfied, providing mutually reinforcing evidence for the target topological phase.

as we require all criteria to be satisfied simultaneously. This definition is rooted in how topological order is

characterized in condensed matter physics: through universal responses and robust phase-diagnostic signatures, not through wavefunction overlap with a reference state. As such, we argue that a single minimum fidelity threshold $\mathcal{F}_{\min}$ is not the appropriate metric for topological phases; the relevant question is whether the prepared state's topological diagnostics are consistent with the target phase.

That said, fidelities remain useful when available: (a) as an internal validation tool for effective-model selection at small sizes, and (b) inform practical ovrhead for the effectiveness of downstream quantum algorithms, as the success probability per trial in eigenstate-projection or quantum phase estimation workflows scales with the fidelity as discussed in the revised Discussion section.

Please also see our response to question 5) about the big-picture level discussions about how this work advances the field.

4) The scaling arguments in the paper are not robust. It is clear from figure 1) that errors will grow with increasing system size. Even more so: six to eight electrons is not enough data to extrapolate. System sizes up to 11 sites is not convincing either. Table 1 shows impressive numbers or two qubit gates, but the ansatz does not entangle all qubits when the system sizes get large, which casts doubts on its viability, or the difficulty of the actual problem.

Authors' reply: We thank the referee for raising this concern and we would like to clarify on a referee's misunderstanding, the label $L_y = 11$ in Fig. 1 represent the circumference of the cylinder, representing a tunable geometric parameter of the cylinder, not the total system size. Our small N_e calculations are used to validate the truncation against our quantitative criteria of fidelity and qualitative criteria of topology and entanglement. The error in our quantum data will be assessed based on the success metric we defined.

We agree with the referee regarding the concern about lightcone and has added the following dedicated paragraph stating the expected circuit depth scaling to larger system size.

> "We interpret the HVA as a digitized adiabatic protocol generated by a local effective Hamiltonian. Lieb–Robinson bounds on the spread of correlations under local dynamics imply an effective light cone with finite velocity. We therefore expect that, for our Laughlin state HVA, the number of repetitions p must grow at least linearly with the system size in order to faithfully reproduce the long-range entanglement structure of the topological phase. As we show below, our ansatz also achieves a linear scaling of total number of variational parameters by generalizing parameters in a HVA layer across the lattice. This avoids the quadratic or worse parameter growth that would result from assigning independent parameters to every microscopic term and aligns with previous work showing that constrained HVA remains expressive while improving trainability."

We appreciate the referee for pointing out this potential confusion regarding Table I. We agree that presenting raw two-qubit gate counts in the main text could be misread as a claim that a fixed-depth circuit suffices at arbitrary size. We therefore moved Table I to the Supplementary Information and removed its surrounding text in the main text to avoid this confusion.

Referee's comment #4:

Issues with regards to the ansatz: The authors are proposing a 5-parameter ansatz for all system sizes. However, that will necessarily create a finite light cone of entanglement. The wave function ansatz will not be able to realize arbitrary long-range entanglement. Even more so, it is likely such ansatz with limited entanglement in many cases can be solved efficiently (or sampled efficiently) on classical hardware.

Authors' reply: We appreciate the referee for raising the question and we have revised the statement regarding number of parameters scaling with system size in the main text. We now distinguish clearly between (i) the per-repetition parameter count, which in an HVA is set by the number of physical generators (in our case, one parameter per $\hat{U}_{km}$ layer) and (ii) the repetitions p of HVA layer. Interpreting HVA as a digitized adiabatic scheme, one choose a small set of operations, U_{km}, and assign a time-dependent schedule β_{km} each.

Discretizing time into p steps yields (# of $\hat{U}_{km}$) $\times p$ parameters. As we demonstrate that including U_{31} will break the formation of a krylov subspace that is significantly smaller than the Hilbert space and restore the correct entanglement area law scaling, this supports that H_{eff} contains enough operations for HVA. Thus we expect the number of operations can be kept constant while depth grows, so the parameter count rises primarily through p.

Conversely a naive, per-term parameterization while scales with the number of Hamiltonian terms (quadratic or worse under common fermionic encodings) is precisely what the HVA was designed to avoid. The original proposal[3] argues explicitly for grouping (generalize) parameters within each physically meaningful operations and warns per-term parameterization is both unrealistic to optimize and typically unnecessary for accuracy. As also demonstrated by Stanisic, et al [1] , few parameter are needed to reproduce key qualitative features of strongly-correlated electronic systems.

We have also included a table of circuit depth vs. (system size, truncation range) in the Supplementary Information (Sec. III.B, page S4, Table S2) to accompany this analysis and as a reference for future works.

Referee's comment #5:

From a big picture perspective I would ask the authors to consider what success would be and how to make advances in the field of fractional quantum hall. It is impossible to determine that from the current manuscript.

Authors' reply: We appreciate the referee for motivating us to articulate the broader impact of this work more clearly. In the revised manuscript, we have revised both the definition of success and the big-picture vision for how this work advances the field. We also refer the referee to our previous detailed discussion in Comment #3 (Question #3) for a fuller explanation of how we define success and its implications for future predictive studies. We summarize the key points below:

1. Our work contribute to the community of Fractional Quantum Hall physics that for the first time on a digital quantum processor, a complete pipeline for simulating material-intrinsic topological order: starting from the physical Hamiltonian of the FQH system, performing a controlled model reduction, constructing a symmetry-preserving Hamiltonian variational ansatz, executing the circuit on trapped-ion hardware with tailored error mitigation, and extracting a suite of phase-diagnostic observables (edge/bulk density structure, correlation functions, and topological entanglement entropy). To the best of our knowledge, there has not been hardware validated demonstration of fermionic, non-stabilizer topological state at this scale.
2. We assess success using phase-diagnostic observables: microscopic liquid structure (bulk-edge density structure and correlation hole) and the universal topological entanglement entropy. These diagnostics remain meaningful when classical benchmarks (e.g., ED/statevector fidelities) are unavailable, because they do not require prior knowledge of the exact ground-state wavefunction.

Building on the workflow demonstrated here, several natural extensions become accessible (stated in the Discussion section of main text):

> "Beyond the Laughlin state, our method can be extended to quasiparticle states as well as to more complex non-Abelian topological order such as the Moore–Read and Read–Rezayi states. The realization of these exotic phases would mark a significant step towards exploring exotic topological phases, providing a robust platform for exploring Abelian and non-Abelian braiding statistics through adiabatic quasiparticle transport, edge and bulk excitations, and nonequilibrium dynamics such as emergent graviton modes in FQH systems."

[3]D. Wecker, M. B. Hastings, and M. Troyer, Progress towards practical quantum variational algorithms, Phys. Rev. A 92, 042303 (2015).

[1]S. Stanisic, J. L. Bosse, F. M. Gambetta, R. A. Santos, W. Mruczkiewicz, T. E. O'Brien, E. Ostby, and A. Montanaro, Observing ground-state properties of the Fermi-Hubbard model using a scalable algorithm on a quantum computer, Nat Commun 13, 5743 (2022).

We also clarify on several outlook directions here:

1. As hardware quality improve to support variational optimization routine on hardware, our pipeline can be applied at larger orbital counts where ED is infeasible, using the observable-centric diagnostic suite established here to explore other states (see next point).
2. Extending the protocol to more filling fractions (e.g., $\nu = 5/2$), where the nature of the ground state (Moore-Read vs. anti-Pfaffian) remains an open question. Our workflow may enable systematic comparison of candidate orders using entanglement- and correlation-based diagnostics.
3. The ability to prepare the Laughlin ground state positions our protocol for studying quasiparticle states and their braiding statistics via adiabatic transport protocols, directly probing the anyonic nature of FQH excitations.

To further establish more concrete outlooks on how our demonstration can enable future progress in Fractional Quantum Hall physics, we added another outlook paragraph emphasizing that our protocol can serve as a practical state-initialization primitive for quantum algorithms whose performance depends strongly on initial-state quality such as phase-estimation and related energy-estimation/filtering workflows. Likewise, imaginary-time and Krylov/Lanczos-type eigenstate-refinement methods require a nonzero (and in practice sufficiently large) overlap with the target low-energy subspace for efficient convergence.

> "Moreover, our protocol is well positioned as a practical state-initialization routine for a broader class of quantum algorithms where high-quality initial states substantially improve algorithms' convergence and practical performance. Taken together, these features position our protocol as a versatile building block for the digital simulation and diagnosis of topological quantum matter."

Referee's comment #6:

Other things to consider:

1) Line 154: The fidelity naturally decays with Ne due to the orthogonality catastrophe of many-body systems [28, 29].

I don't consider this a useful statement. Whether or not the orthogonality catastrophe is important, the authors are using small system sizes with fixed number of variational parameters. The idea of the orthogonality catastrophe is not important for this situation at these system sizes.

Authors' reply: We appreciate the referee for pointing out this confusion as our intent was not to suggest the connection between orthogonality catastrophe and the ansatz but only to note decreasing wavefunction overlap behavior in Fig.1. We have removed this sentence for clarity.

2) Line 245: Optimizing $|\psi(\beta_j)\rangle_{\text{eff}}$ with larger system size did not achieve higher fidelity (see Supplementary Information), indicating that the accumulation of infidelity is mainly due to the orthogonality catastrophe [28, 29].

I don't think I even understand this statement. What does the orthogonality catastrophe have to do with this observation.

Authors' reply: We thank the referee for raising this confusion. The observation we wish to convey is: our HVA construction protocol and end-to-end simulation workflow pipeline provide resilience towards trainability issues. Parameters we found on a small instance remain near-optimal when transferred to larger systems, a phenomenon studied and shown to mitigate barren plateaus in practice[4]. We have removed the mention of orthogonality catastrophe and revised the writing to bring out this point explicitly.

> "Optimizing $|\psi(\{\beta_j\})\rangle_{\text{eff}}$ with larger system size did not achieve higher fidelity (see Supplementary Information), further supporting parameter transferability and our construction's resilience

[4] A. A. Mele, G. B. Mbeng, G. E. Santoro, M. Collura, and P. Torta, Avoiding barren plateaus via transferability of smooth solutions in a Hamiltonian variational ansatz, Phys. Rev. A 106, L060401 (2022).

to barren plateau."

3) Line 421: In addition, our work introduces a new quantum methodology for studying strongly correlated topological materials.

My understanding of this work is that you are using an HVA like ansatz which is not even being optimized on quantum hardware. What is the "new quantum methodology"?

Authors' reply: We believe this question has been addressed in comment #2.

4) Line 160: the effective Hamiltonian HTT forms a Krylov subspace K, an example of Hilbert space fragmentation [30], which can be used to map FQH model under TT

I didn't understand this point (or the rest of this paragraph). A Hamiltonian does not form a Kyrlov subspace as far as I know. The application of a Hamiltonian to a wavefunction multiple times can create a Kyrlov subspace. Could the authors expand or improve the writing in this paragraph?

Authors' reply: We thank the referee for pointing out this issue. We have improved the writing of the specific paragraph to clearly define the Krylov subspace we are referring to.

> "With only the lowest-order scattering ($k + m \leq 3$) included, the action of the effective Hamiltonian H_{TT} on the CDW state $|\Psi_{\mathrm{CDW}}\rangle$ forms a Krylov subspace $\mathcal{K}(H_{\mathrm{TT}}, |\Psi_{\mathrm{CDW}}\rangle)$. "

Referee #1

Referee's comments:

The authors have made a genuine effort addressing my (admittedly quite demanding and critical) comments. I think among all of the studies that prepare states on current quantum computing hardware which are classically solvable, this is among the best-executed. In particular, the determination of the top. entanglement entropy is an impressive demonstration.

I still have reservations about the scalability of this approach. VQE is intrinsically very challenging to scale. However, in the present study, the authors present several noteworthy techniques to reduce the overhead which make it not impossible to think that this specific approach may be more scalable than usual VQE studies. I do agree with the author's rebuttal of my comments in that a combination of all of these existing techniques is actually a nontrivial feat.

Authors' reply:

We thank the reviewer for the careful assessment and for recommending publication. We also appreciate the perspective on scalability and the recognition of the end-to-end implementation as a nontrivial advance.

Referee #2

Referee's comments:

After reading the revised manuscript and replies to the reports, I have concluded this work is suitable for publication in Nature Communications. The authors have substantially improved the clarity of the work and its novelty through their rewrites as well as its impact by including additional calculations to confirm the nature of the state.

There is only one point I would request be clarified throughout the text. I believe when the authors refer to the exact and Laughlin state, they mean the same state obtained via exact diagonalization. This mixing of terminology is a bit confusing as sometimes it makes one think they are using the trial wave function. I recommend that the authors refer to this only as the exact state throughout and mention briefly upon introducing the state that it is known to match very well with the Laughlin trial wave function.

Authors' reply:

We thank the reviewer for the positive evaluation and for the helpful suggestion on terminology. We have revised the manuscript to remove ambiguity, adding a brief statement to clarify

> "the $\nu = 1/3$ Laughlin state (referred to as exact state throughout this work) is an exact ground state."

This change is applied consistently throughout the main text and Supplementary Information.

Referee #3

Referee's comment #1:

I co-reviewed this manuscript with one of the reviewers who provided the listed reports. This is part of the Nature Communications initiative to facilitate training in peer review and to provide appropriate recognition for Early Career Researchers who co-review manuscripts.

Authors' reply:

We thank the referee for their time and thoughtful contribution to the co-review process in both rounds of evaluation. We appreciate their engagement with the manuscript and their role in the peer-review process.
